# 2016 AHA/ACC Guideline on the Management of Patients With Lower Extremity Peripheral Artery Disease: Executive Summary: A Report of the American College of Cardiology/American Heart Association Task Force on Clinical Practice Guidelines

**DOI:** 10.1161/CIR.0000000000000470

**Published:** 2016-11-13

**Authors:** Writing Committee Members, Marie D. Gerhard-Herman, Heather L. Gornik, Coletta Barrett, Neal R. Barshes, Matthew A. Corriere, Douglas E. Drachman, Lee A. Fleisher, Francis Gerry R. Fowkes, Naomi M. Hamburg, Scott Kinlay, Robert Lookstein, Sanjay Misra, Leila Mureebe, Jeffrey W. Olin, Rajan A.G. Patel, Judith G. Regensteiner, Andres Schanzer, Mehdi H. Shishehbor, Kerry J. Stewart, Diane Treat-Jacobson, M. Eileen Walsh, Jonathan L. Halperin, ACC/AHA Task Force on Clinical Practice Guidelines

**Affiliations:** * Writing committee members are required to recuse themselves from voting on sections to which their specific relationships with industry and other entities may apply; see Appendix 1 for recusal information; † Functioning as the lay volunteer/patient representative; ‡ ACC/AHA Representative; § Vascular and Endovascular Surgery Society Representative; ‖ Society for Cardiovascular Angiography and Interventions Representative; ¶ ACC/AHA Task Force on Clinical Practice Guidelines Liaison; # Inter-Society Consensus for the Management of Peripheral Arterial Disease Representative; ** Society for Vascular Medicine Representative; †† Society of Interventional Radiology Representative; ‡‡ Society for Clinical Vascular Surgery Representative; §§ Society for Vascular Surgery Representative; ‖‖ American Association of Cardiovascular and Pulmonary Rehabilitation Representative; ¶¶ Society for Vascular Nursing RepresentativeChair, ACC/AHA Task Force on Clinical Practice Guidelines

**Keywords:** AHA Scientific Statements, peripheral artery disease, claudication, critical limb ischemia, acute limb ischemia, antiplatelet agents, supervised exercise, endovascular procedures, bypass surgery, limb salvage, smoking cessation

## Abstract

Since 1980, the American College of Cardiology (ACC) and American Heart Association (AHA) have translated scientific evidence into clinical practice guidelines with recommendations to improve cardiovascular health. These guidelines, based on systematic methods to evaluate and classify evidence, provide a cornerstone of quality cardiovascular care.

In response to reports from the Institute of Medicine^1,2^ and a mandate to evaluate new knowledge and maintain relevance at the point of care, the ACC/AHA Task Force on Clinical Practice Guidelines (Task Force) modified its methodology.^3–5^ The relationships among guidelines, data standards, appropriate use criteria, and performance measures are addressed elsewhere.^5^

**Intended Use:**

Practice guidelines provide recommendations applicable to patients with or at risk of developing cardiovascular disease. The focus is on medical practice in the United States, but guidelines developed in collaboration with other organizations may have a broader target. Although guidelines may be used to inform regulatory or payer decisions, the intent is to improve quality of care and align with patients' interests. Guidelines are intended to define practices meeting the needs of patients in most, but not all, circumstances, and should not replace clinical judgment. Guidelines are reviewed annually by the Task Force and are official policy of the ACC and AHA. Each guideline is considered current until it is updated, revised, or superseded by published addenda, statements of clarification, focused updates, or revised full-text guidelines. To ensure that guidelines remain current, new data are reviewed biannually to determine whether recommendations should be modified. In general, full revisions are posted in 5-year cycles.^3–6^

**Modernization:**

Processes have evolved to support the evolution of guidelines as “living documents” that can be dynamically updated. This process delineates a recommendation to address a specific clinical question, followed by concise text (ideally <250 words) and hyperlinked to supportive evidence. This approach accommodates time constraints on busy clinicians and facilitates easier access to recommendations via electronic search engines and other evolving technology.

**Evidence Review:**

Writing committee members review the literature; weigh the quality of evidence for or against particular tests, treatments, or procedures; and estimate expected health outcomes. In developing recommendations, the writing committee uses evidence-based methodologies that are based on all available data.^3–7^ Literature searches focus on randomized controlled trials (RCTs) but also include registries, nonrandomized comparative and descriptive studies, case series, cohort studies, systematic reviews, and expert opinion. Only selected references are cited.

The Task Force recognizes the need for objective, independent Evidence Review Committees (ERCs) that include methodologists, epidemiologists, clinicians, and biostatisticians who systematically survey, abstract, and assess the evidence to address systematic review questions posed in the PICOTS format (P=population, I=intervention, C=comparator, O=outcome, T=timing, S=setting).^2,4–6^ Practical considerations, including time and resource constraints, limit the ERCs to evidence that is relevant to key clinical questions and lends itself to systematic review and analysis that could affect the strength of corresponding recommendations.

**Guideline-Directed Management and Treatment:**

The term “guideline-directed management and therapy” (GDMT) refers to care defined mainly by ACC/AHA Class I recommendations. For these and all recommended drug treatment regimens, the reader should confirm dosage with product insert material and carefully evaluate for contraindications and interactions. Recommendations are limited to treatments, drugs, and devices approved for clinical use in the United States.

**Class of Recommendation and Level of Evidence:**

The Class of Recommendation (COR; ie, the strength of the recommendation) encompasses the anticipated magnitude and certainty of benefit in proportion to risk. The Level of Evidence (LOE) rates evidence supporting the effect of the intervention on the basis of the type, quality, quantity, and consistency of data from clinical trials and other reports (Table 1).^3–5^ Unless otherwise stated, recommendations are sequenced by COR and then by LOE. Where comparative data exist, preferred strategies take precedence. When >1 drug, strategy, or therapy exists within the same COR and LOE and no comparative data are available, options are listed alphabetically.

**Relationships With Industry and Other Entities:**

The ACC and AHA sponsor the guidelines without commercial support, and members volunteer their time. The Task Force zealously avoids actual, potential, or perceived conflicts of interest that might arise through relationships with industry or other entities (RWI). All writing committee members and reviewers are required to disclose current industry relationships or personal interests, from 12 months before initiation of the writing effort. Management of RWI involves selecting a balanced writing committee and assuring that the chair and a majority of committee members have no relevant RWI (Appendix 1). Members are restricted with regard to writing or voting on sections to which their RWI apply. For transparency, members' comprehensive disclosure information is available online. Comprehensive disclosure information for the Task Force is also available online.

The Task Force strives to avoid bias by selecting experts from a broad array of backgrounds representing different geographic regions, sexes, ethnicities, intellectual perspectives/biases, and scopes of clinical practice, and by inviting organizations and professional societies with related interests and expertise to participate as partners or collaborators.

**Individualizing Care in Patients With Associated Conditions and Comorbidities:**

Managing patients with multiple conditions can be complex, especially when recommendations applicable to coexisting illnesses are discordant or interacting.^8^ The guidelines are intended to define practices meeting the needs of patients in most, but not all, circumstances. The recommendations should not replace clinical judgment.

**Clinical Implementation:**

Management in accordance with guideline recommendations is effective only when followed. Adherence to recommendations can be enhanced by shared decision making between clinicians and patients, with patient engagement in selecting interventions on the basis of individual values, preferences, and associated conditions and comorbidities. Consequently, circumstances may arise in which deviations from these guidelines are appropriate.

The reader is encouraged to consult the full-text guideline^9^ for additional guidance and details with regard to lower extremity peripheral artery disease (PAD) because the executive summary contains limited information.

## 1. Introduction

### 1.1. Methodology and Evidence Review

The recommendations listed in this guideline are, whenever possible, evidence based. An initial extensive evidence review, which included literature derived from research involving human subjects, published in English, and indexed in MEDLINE (through PubMed), EMBASE, the Cochrane Library, the Agency for Healthcare Research and Quality, and other selected databases relevant to this guideline, was conducted from January through September 2015. Key search words included but were not limited to the following: *acute limb ischemia, angioplasty, ankle-brachial index, anticoagulation, antiplatelet therapy, atypical leg symptoms, blood pressure lowering/hypertension, bypass graft/bypass grafting/surgical bypass, cilostazol, claudication/intermittent claudication, critical limb ischemia/severe limb ischemia, diabetes, diagnostic testing, endovascular therapy, exercise rehabilitation/exercise therapy/exercise training/ supervised exercise, lower extremity/foot wound/ulcer, peripheral artery disease/peripheral arterial disease/ peripheral vascular disease/lower extremity arterial disease, smoking/smoking cessation, statin, stenting, and vascular surgery*. Additional relevant studies published through September 2016, during the guideline writing process, were also considered by the writing committee, and added to the evidence tables when appropriate. The final evidence tables included in the Online Data Supplement summarize the evidence utilized by the writing committee to formulate recommendations. Additionally, the writing committee reviewed documents related to lower extremity PAD previously published by the ACC and AHA.^10,11^ References selected and published in this document are representative and not all-inclusive.

As stated in the Preamble, the ACC/AHA guideline methodology provides for commissioning an independent ERC to address systematic review questions (PICOTS format) to inform recommendations developed by the writing committee. All other guideline recommendations (not based on the systematic review questions) were also subjected to an extensive evidence review process. For this guideline, the writing committee in conjunction with the Task Force and ERC Chair identified the following systematic review questions: 1) Is antiplatelet therapy beneficial for prevention of cardiovascular events in the patient with symptomatic or asymptomatic lower extremity PAD? 2) What is the effect of revascularization, compared with optimal medical therapy and exercise training, on functional outcome and quality of life (QoL) among patients with claudication? Each question has been the subject of recently published, systematic evidence reviews.^12–14^ The quality of these evidence reviews was appraised by the ACC/AHA methodologist and a vendor contracted to support this process (Doctor Evidence [Santa Monica, CA]). Few substantive randomized or nonrandomized studies had been published after the end date of the literature searches used for the existing evidence reviews, so the ERC concluded that no additional systematic review was necessary to address either of these critical questions.

A third systematic review question was then identified: 3) Is one revascularization strategy (endovascular or surgical) associated with improved cardiovascular and limb-related outcomes in patients with critical limb ischemia (CLI)? This question had also been the subject of a high-quality systematic review that synthesized evidence from observational data and an RCT^15^; additional RCTs addressing this question are ongoing.^16–18^ The writing committee and the Task Force decided to expand the survey to include more relevant randomized and observational studies. Based on evaluation of this additional evidence the ERC decided that further systematic review was not needed to inform the writing committee on this question. Hence, the ERC and writing committee concluded that available systematic reviews could be used to inform the development of recommendations addressing each of the 3 systematic review questions specified above. The members of the Task Force and writing committee thank the members of the ERC that began this process and their willingness to participate in this volunteer effort. They include Aruna Pradhan, MD, MPH (ERC Chair); Natalie Evans, MD; Peter Henke, MD; Dharam J. Kumbhani, MD, SM, FACC; and Tamar Polonsky, MD.

### 1.2. Organization of the Writing Committee

The writing committee consisted of clinicians, including noninvasive and interventional cardiologists, exercise physiologists, internists, interventional radiologists, vascular nurses, vascular medicine specialists, and vascular surgeons, as well as clinical researchers in the field of vascular disease, a nurse (in the role of patient representative), and members with experience in epidemiology and/or health services research. The writing committee included representatives from the ACC and AHA, American Association of Cardiovascular and Pulmonary Rehabilitation, Inter-Society Consensus for the Management of Peripheral Arterial Disease, Society for Cardiovascular Angiography and Interventions, Society for Clinical Vascular Surgery, Society of Interventional Radiology, Society for Vascular Medicine, Society for Vascular Nursing, Society for Vascular Surgery, and Vascular and Endovascular Surgery Society.

### 1.3. Document Review and Approval

This document was reviewed by 2 official reviewers nominated by the ACC and AHA; 1 to 2 reviewers each from the American Association of Cardiovascular and Pulmonary Rehabilitation, Inter-Society Consensus for the Management of Peripheral Arterial Disease, Society for Cardiovascular Angiography and Interventions, Society for Clinical Vascular Surgery, Society of Interventional Radiology, Society for Vascular Medicine, Society for Vascular Nursing, Society for Vascular Surgery, and Vascular and Endovascular Surgery Society; and 16 additional individual content reviewers. Reviewers' RWI information was distributed to the writing committee and is published in this document (Appendix 2).

This document was approved for publication by the governing bodies of the ACC and the AHA and endorsed by the American Association of Cardiovascular and Pulmonary Rehabilitation, Inter-Society Consensus for the Management of Peripheral Arterial Disease, Society for Cardiovascular Angiography and Interventions, Society for Clinical Vascular Surgery, Society of Interventional Radiology, Society for Vascular Medicine, Society for Vascular Nursing, Society for Vascular Surgery, and Vascular and Endovascular Surgery Society.

### 1.4. Scope of Guideline

Lower extremity PAD is a common cardiovascular disease that is estimated to affect approximately 8.5 million Americans above the age of 40 years and is associated with significant morbidity, mortality, and QoL impairment.^19^ It has been estimated that 202 million people worldwide have PAD.^20^ The purpose of this document is to provide a contemporary guideline for diagnosis and management of patients with lower extremity PAD. This document supersedes recommendations related to lower extremity PAD in the “ACC/AHA 2005 Guidelines for the Management of Patients With Peripheral Arterial Disease”^10^ and the “2011 ACCF/AHA Focused Update of the Guideline for the Management of Patients With Peripheral Artery Disease.”^11^ The scope of this guideline is limited to atherosclerotic disease of the lower extremity arteries (PAD) and includes disease of the aortoiliac, femoropopliteal, and infrapopliteal arterial segments. It does not address nonatherosclerotic causes of lower extremity arterial disease, such as vasculitis, fibromuscular dysplasia, physiological entrapment syndromes, cystic adventitial disease, and other entities. Future guidelines will address aneurysmal disease of the abdominal aorta and lower extremity arteries and diseases of the renal and mesenteric arteries.

For the purposes of this guideline, key terms associated with PAD are defined in Table 2.

## 2. Clinical Assessment For Pad

Evaluating the patient at increased risk of PAD (Table 3) begins with the clinical history, review of symptoms, and physical examination. The symptoms and signs of PAD are variable. Patients with PAD may experience the classic symptom of claudication or may present with advanced disease, including CLI. Studies have demonstrated that the majority of patients with confirmed PAD do not have typical claudication but have other non-joint-related limb symptoms (atypical leg symptoms) or are asymptomatic.^40,41^ Patients with PAD who have atypical leg symptoms or no symptoms may have functional impairment comparable to patients with claudication.^42^ The vascular examination for PAD includes pulse palpation, auscultation for femoral bruits, and inspection of the legs and feet. Lower extremity pulses are assessed and rated as follows: 0, absent; 1, diminished; 2, normal; or 3, bounding. See Table 4 for history and physical examination findings suggestive of PAD. To confirm the diagnosis of PAD, abnormal physical examination findings must be confirmed with diagnostic testing (Section 3), generally with the ankle-brachial index (ABI) as the initial test.

Patients with confirmed diagnosis of PAD are at increased risk for subclavian artery stenosis.^43–45^ An inter-arm blood pressure difference of >15 to 20 mm Hg is abnormal and suggestive of subclavian (or innominate) artery stenosis. Measuring blood pressure in both arms identifies the arm with the highest systolic pressure, a requirement for accurate measurement of the ABI.^46^ Identification of unequal blood pressures in the arms also allows for more accurate measurement of blood pressure in the treatment of hypertension (ie, blood pressure is taken at the arm with higher measurements).

See Online Data Supplements 1 and 2 for data supporting Section 2.

### 2.1. History and Physical Examination: Recommendations


**Recommendations for History and Physical Examination**
**COR****LOE****Recommendations**
**I****B-NR**Patients at increased risk of PAD (Table 3) should undergo a comprehensive medical history and a review of symptoms to assess for exertional leg symptoms, including claudication or other walking impairment, ischemic rest pain, and nonhealing wounds.^40–42,47–49^
**I****B-NR**Patients at increased risk of PAD (Table 3) should undergo vascular examination, including palpation of lower extremity pulses (ie, femoral, popliteal, dorsalis pedis, and posterior tibial), auscultation for femoral bruits, and inspection of the legs and feet.^48,50,51^
**I****B-NR**Patients with PAD should undergo noninvasive blood pressure measurement in both arms at least once during the initial assessment.^43–45^


## 3. Diagnostic Testing For The Patient With Suspected Lower Extremity Pad (Claudication or Cli): Recommendations

History or physical examination findings suggestive of PAD need to be confirmed with diagnostic testing. The resting ABI is the initial diagnostic test for PAD and may be the only test required to establish the diagnosis and institute GDMT The resting ABI is a simple, noninvasive test that is obtained by measuring systolic blood pressures at the arms (brachial arteries) and ankles (dorsalis pedis and posterior tibial arteries) in the supine position by using a Doppler device. The ABI of each leg is calculated by dividing the higher of the dorsalis pedis pressure or posterior tibial pressure by the higher of the right or left arm blood pressure.^46^ Segmental lower extremity blood pressures and Doppler or plethysmographic waveforms (pulse volume recordings) are often performed along with the ABI and can be used to localize anatomic segments of disease (eg, aortoiliac, femoropopliteal, infrapopliteal).^22,53,54^

Depending on the clinical presentation (eg, claudication or CLI) and the resting ABI values, additional physiological testing studies may be indicated, including exercise treadmill ABI testing, measurement of the toe-brachial index (TBI), and additional perfusion assessment measures (eg, transcutaneous oxygen pressure [TcPO_2_], or skin perfusion pressure [SPP]). Exercise treadmill ABI testing is important to objectively measure functional limitations attributable to leg symptoms and is useful in establishing the diagnosis of lower extremity PAD in the symptomatic patient when resting ABIs are normal or borderline.^54–59^ The TBI is used to establish the diagnosis of PAD in the setting of non-compressible arteries (ABI >1.40) and may also be used to assess perfusion in patients with suspected CLI. Studies for anatomic imaging assessment (duplex ultrasound, computed tomography angiography [CTA], or magnetic resonance angiography [MRA], invasive angiography) are generally reserved for highly symptomatic patients in whom revascularization is being considered. Depending on the modality, these studies may confer procedural risk.

**Figure 1:**
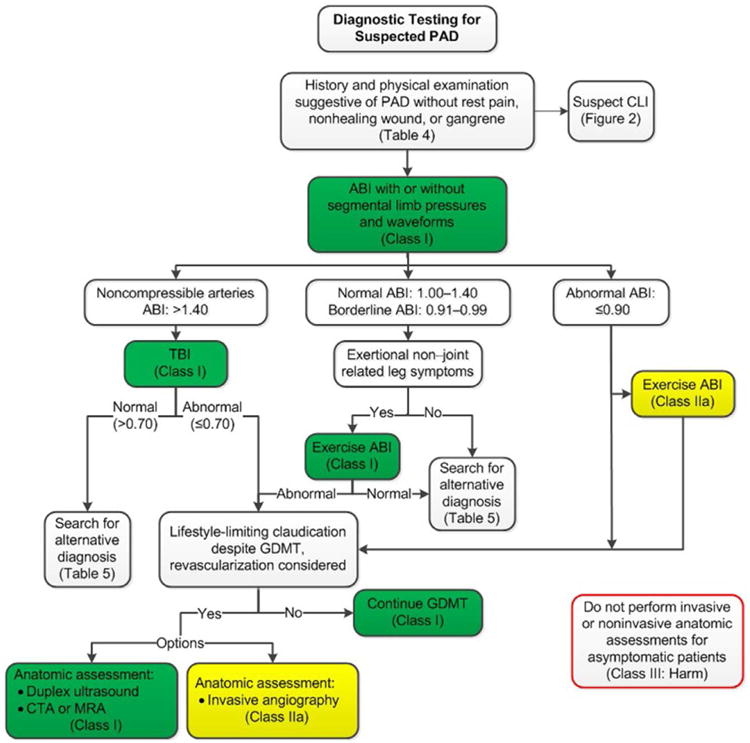
Diagnostic Testing for Suspected PAD Colors correspond to Class of Recommendation in Table 1. ABI indicates ankle-brachial index; CLI, critical limb ischemia; CTA, computed tomography angiography; GDMT, guideline-directed management and therapy; MRA, magnetic resonance angiography; PAD, peripheral artery disease; and TBI, toe-brachial index.

**Figure 2:**
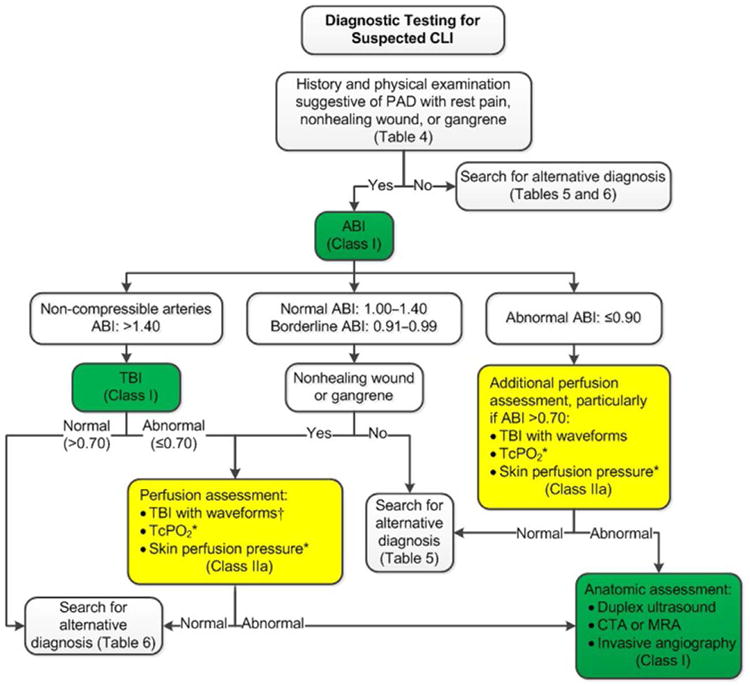
Diagnostic Testing for Suspected CLI Colors correspond to Class of Recommendation in Table 1. *Order based on expert consensus. †TBI with waveforms, if not already performed. ABI indicates ankle-brachial index; CLI, critical limb ischemia; CTA, computed tomography angiography; MRA, magnetic resonance angiography; TcPO_2_, transcutaneous oxygen pressure; and TBI, toe-brachial index.

See Table 5 for alternative causes of leg pain in the patient with normal ABI and physiological testing; Figure 1 for the algorithm on diagnostic testing for suspected PAD and claudication; Table 6 for alternative causes of nonhealing wounds in patients without PAD; Figure 2 for the algorithm on diagnostic testing for suspected CLI; and Online Data Supplements 3 to 7 for data supporting Section 3.

### 3.1. Resting ABI for Diagnosing PAD


**Recommendations for Resting ABI for Diagnosing PAD**
**COR****LOE****Recommendations**
**I****B-NR**In patients with history or physical examination findings suggestive of PAD (Table 4), the resting ABI, with or without segmental pressures and waveforms, is recommended to establish the diagnosis.^60–65^
**I****C-LD**Resting ABI results should be reported as abnormal (ABI ≤0.90), borderline (ABI 0.91–0.99), normal (1.00–1.40), or noncompressible (ABI >1.40).^46,63–66^
**IIa****B-NR**In patients at increased risk of PAD (Table 3) but without history or physical examination findings suggestive of PAD (Table 4), measurement of the resting ABI is reasonable.^41,42,67–89^
**III: No Benefit****B-NR**In patients not at increased risk of PAD (Table 3) and without history or physical examination findings suggestive of PAD (Table 4), the ABI is not recommended.^87,90^


### 3.2. Physiological Testing


**Recommendations for Physiological Testing**
**COR****LOE****Recommendations**
**I****B-NR**Toe-brachial index (TBI) should be measured to diagnose patients with suspected PAD when the ABI is greater than 1.40.^66,91–94^
**I****B-NR**Patients with exertional non–joint-related leg symptoms and normal or borderline resting ABI (>0.90 and ≤1.40) should undergo exercise treadmill ABI testing to evaluate for PAD.^54–59^
**IIa****B-NR**In patients with PAD and an abnormal resting ABI (≤0.90), exercise treadmill ABI testing can be useful to objectively assess functional status.^54–59^
**IIa****B-NR**In patients with normal (1.00–1.40) or borderline (0.91–0.99) ABI in the setting of nonhealing wounds or gangrene, it is reasonable to diagnose CLI by using TBI with waveforms, TcPO_2_, or SPP.^95–99^
**IIa****B-NR**In patients with PAD with an abnormal ABI (≤0.90) or with noncompressible arteries (ABI >1.40 and TBI ≤0.70) in the setting of nonhealing wounds or gangrene, TBI with waveforms, TcPO_2_, or SPP can be useful to evaluate local perfusion.^95–99^


### 3.3. Imaging for Anatomic Assessment


**Recommendations for Imaging for Anatomic Assessment**
**COR****LOE****Recommendations**
**I****B-NR**Duplex ultrasound, CTA, or MRA of the lower extremities is useful to diagnose anatomic location and severity of stenosis for patients with symptomatic PAD in whom revascularization is considered.^100–103^
**I****C-EO**Invasive angiography is useful for patients with CLI in whom revascularization is considered.
**IIa****C-EO**Invasive angiography is reasonable for patients with lifestyle-limiting claudication with an inadequate response to GDMT for whom revascularization is considered.
**III: Harm****B-R**Invasive and noninvasive angiography (ie, CTA, MRA) should not be performed for the anatomic assessment of patients with asymptomatic PAD.^104–106^


## 4. Screening For Atherosclerotic Disease In Other Vascular Beds For The Patient With Pad: Recommendations

See Online Data Supplement 8 for data supporting Section 4.

### 4.1. Abdominal Aortic Aneurysm

PAD has been recognized as a risk factor for abdominal aortic aneurysm (AAA). In observational studies, the prevalence of AAA (aortic diameter ≥3 cm) was higher in patients with symptomatic PAD than in the general population^107,108^ and in a population of patients with atherosclerotic risk factors.^109^ The prevalence of AAA among patients with PAD increased with age, beginning in patients ≥55 years of age, and was highest in patients ≥75 years of age.^107^ There are no data on AAA screening in patients with asymptomatic PAD. This section refers to screening patients with symptomatic PAD for AAA. Recommendations for screening the general population with risk factors for AAA (based on age, sex, smoking history, and family history) have been previously published.^10^


**Recommendation for Abdominal Aortic Aneurysm**
**COR****LOE****Recommendation**
**IIa****B-NR**A screening duplex ultrasound for AAA is reasonable in patients with symptomatic PAD.^107–109^


### 4.2. Screening for Asymptomatic Atherosclerosis in Other Arterial Beds (Coronary, Carotid, and Renal Arteries)

The prevalence of atherosclerosis in the coronary, carotid, and renal arteries is higher in patients with PAD than in those without PAD.^109–115^ However, intensive atherosclerosis risk factor modification in patients with PAD is justified regardless of the presence of disease in other arterial beds. Thus, the only justification for screening for disease in other arterial beds is if revascularization results in a reduced risk of myocardial infarction (MI), stroke, or death, and this has never been shown. Currently, there is no evidence to demonstrate that screening all patients with PAD for asymptomatic atherosclerosis in other arterial beds improves clinical outcome. Intensive treatment of risk factors through GDMT is the principle method for preventing adverse cardiovascular ischemic events from asymptomatic disease in other arterial beds.

## 5. Medical Therapy For The Patient With Pad: Recommendations

Patients with PAD should receive a comprehensive program of GDMT, including structured exercise and lifestyle modification, to reduce cardiovascular ischemic events and improve functional status. Smoking cessation is a vital component of care for patients with PAD who continue to smoke. A guideline-based program of pharmacotherapy to reduce cardiovascular ischemic events and limb-related events should be prescribed for each patient with PAD and is customized to individual risk factors, such as whether the patient also has diabetes mellitus. Pharmacotherapy for the patient with PAD includes antiplatelet and statin agents and is customized to additional risk factors, such as whether the patient also has diabetes mellitus or hypertension. Previous studies have demonstrated that patients with PAD are less likely to receive GDMT than patients with other forms of cardiovascular disease, including coronary artery disease.^116–118^ Cilostazol is an effective medical therapy for treatment of leg symptoms and walking impairment due to claudication.^119^ However, side effects include headache, diarrhea, dizziness, and palpitations and in 1 trial, 20% of patients discontinued cilostazol within 3 months.^120^

See Online Data Supplements 13 to 19 for data supporting Section 5.

### 5.1. Antiplatelet, Statin, Antihypertensive Agents, and Oral Anticoagulation


**Recommendations for Antiplatelet, Statin, and Antihypertensive Agents**
**COR****LOE****Recommendations**
Antiplatelet Agents
**I****A**Antiplatelet therapy with aspirin alone (range 75–325 mg per day) or clopidogrel alone (75 mg per day) is recommended to reduce MI, stroke, and vascular death in patients with symptomatic PAD.^121–124^
**IIa****C-EO**In asymptomatic patients with PAD (ABI ≤0.90), antiplatelet therapy is reasonable to reduce the risk of MI, stroke, or vascular death.
**IIb****B-R**In asymptomatic patients with borderline ABI (0.91–0.99), the usefulness of antiplatelet therapy to reduce the risk of MI, stroke, or vascular death is uncertain.^67,68^
**IIb****B-R**The effectiveness of dual antiplatelet therapy (aspirin and clopidogrel) to reduce the risk of cardiovascular ischemic events in patients with symptomatic PAD is not well established.^125,126^
**IIb****C-LD**Dual antiplatelet therapy (aspirin and clopidogrel) may be reasonable to reduce the risk of limb-related events in patients with symptomatic PAD after lower extremity revascularization.^127–130^
**IIb****B-R**The overall clinical benefit of vorapaxar added to existing antiplatelet therapy in patients with symptomatic PAD is uncertain.^131–134^
Statin Agents
**I****A**Treatment with a statin medication is indicated for all patients with PAD.^88,135–139^
Antihypertensive Agents
**I****A**Antihypertensive therapy should be administered to patients with hypertension and PAD to reduce the risk of MI, stroke, heart failure, and cardiovascular death.^140–144^
**IIa****A**The use of angiotensin-converting enzyme inhibitors or angiotensin-receptor blockers can be effective to reduce the risk of cardiovascular ischemic events in patients with PAD.^143,145,146^
Oral Anticoagulation
**IIb****B-R**The usefulness of anticoagulation to improve patency after lower extremity autogenous vein or prosthetic bypass is uncertain.^147–149^
**III: Harm****A**Anticoagulation should not be used to reduce the risk of cardiovascular ischemic events in patients with PAD.^148,150–152^


### 5.2. Smoking Cessation


**Recommendations for Smoking Cessation**
**COR****LOE****Recommendations**
**I****A**Patients with PAD who smoke cigarettes or use other forms of tobacco should be advised at every visit to quit.^153–155^
**I****A**Patients with PAD who smoke cigarettes should be assisted in developing a plan for quitting that includes pharmacotherapy (ie, varenicline, bupropion, and/or nicotine replacement therapy) and/or referral to a smoking cessation program.^153,156–158^
**I****B-NR**Patients with PAD should avoid exposure to environmental tobacco smoke at work, at home, and in public places.^159,160^


### 5.3. Glycemic Control


**Recommendations for Glycemic Control**
**COR****LOE****Recommendations**
**I****C-EO**Management of diabetes mellitus in the patient with PAD should be coordinated between members of the healthcare team.
**IIa****B-NR**Glycemic control can be beneficial for patients with CLI to reduce limb-related outcomes^161,162^


### 5.4. Cilostazol, Pentoxifylline, and Chelation Therapy


**Recommendations for Cilostazol, Pentoxifylline, and Chelation Therapy**
**COR****LOE****Recommendations**
**I****A**Cilostazol is an effective therapy to improve symptoms and increase walking distance in patients with claudication.^119,163^
**III: No Benefit****B-R**Pentoxifylline is not effective for treatment of claudication.^119,164^
**III: No Benefit****B-R**Chelation therapy (eg, ethylenediaminetetraacetic acid) is not beneficial for treatment of claudication.^165^


### 5.5. Homocysteine Lowering


**Recommendation for Homocysteine Lowering**
**COR****LOE****Recommendation**
**III: No Benefit****B-R**B-complex vitamin supplementation to lower homocysteine levels for prevention of cardiovascular events in patients with PAD is not recommended.^166–168^


### 5.6. Infuenza Vaccination


**Recommendation for Influenza Vaccination**
**COR****LOE****Recommendation**
**I****C-EO**Patients with PAD should have an annual influenza vaccination.


## 6. Structured Exercise Therapy: Recommendations

Structured exercise therapy is an important element of care for the patient with PAD. Components of structured exercise programs for PAD are outlined in Table 7. The data supporting the efficacy of supervised exercise programs as an initial treatment for claudication continue to develop and remain convincing, building on many earlier RCTs.^28–34,36,169,170^ Trials with long-term follow-up from 18 months^25,26^ to 7 years^24^ have demonstrated a persistent benefit of supervised exercise in patients with claudication. The risk–benefit ratio for supervised exercise in PAD is favorable, with an excellent safety profile in patients screened for absolute contraindications to exercise such as exercise-limiting cardiovascular disease, amputation or wheelchair confinement, and other major comorbidities that would preclude exercise.^24,27,37,171–174^

Studies supporting structured community- or home-based programs for patients with PAD are more recent than studies supporting supervised exercise programs and have provided strong evidence in support of the community- or home-based approach.^35,37,39,80,86,171^ Unstructured community- or home-based walking programs that consist of providing general recommendations to patients with claudication to simply walk more are not efficacious.^38^

See Online Data Supplements 32 and 33 for data supporting Section 6.


**Recommendations for Structured Exercise Therapy**
**COR****LOE****Recommendations**
**I****A**In patients with claudication, a supervised exercise program is recommended to improve functional status and QoL and to reduce leg symptoms.^24–26,28–34,36,169,170^
**I****B-R**A supervised exercise program should be discussed as a treatment option for claudication before possible revascularization.^24–26^
**IIa****A**In patients with PAD, a structured community-or home-based exercise program with behavioral change techniques can be beneficial to improve walking ability and functional status.^37,80,86,171^
**IIa****A**In patients with claudication, alternative strategies of exercise therapy, including upper-body ergometry, cycling, and pain-free or low-intensity walking that avoids moderate-to-maximum claudication while walking, can be beneficial to improve walking ability and functional status.^27,173,175,176^


## 7. Minimizing Tissue Loss In Patients With Pad: Recommendations

Prevention of wounds through patient education, foot examination, and prompt recognition of foot infection is important to minimize tissue loss among patients with PAD. Education includes teaching patients about healthy foot behaviors (eg, daily inspection of feet, wearing of shoes and socks; avoidance of barefoot walking), the selection of proper footwear, and the importance of seeking medical attention for new foot problems.^177^ Educational efforts are especially important for patients with PAD who have diabetes mellitus with peripheral neuropathy.

Foot infections (infection of any of the structures distal to the malleoli) may include cellulitis, abscess, fasciitis, tenosynovitis, septic joint space infection, and osteomyelitis. Because of the consequences associated with untreated foot infection—especially in the presence of PAD—clinicians should maintain a high index of suspicion.^178^ Foot infection is suspected if the patient presents with local pain or tenderness; periwound erythema; periwound edema, induration, or fluctuance; pretibial edema; any discharge (especially purulent); foul odor; visible bone or a wound that probes to bone; or signs of a systemic inflammatory response (including temperature >38°C or <36°C, heart rate >90/min, respiratory rate >20/min or Paco_2_ <32 mm Hg, white blood cell count >12 000 or <4000/mcL or >10% immature forms).^179^ It is recognized that the presence of diabetes mellitus with peripheral neuropathy and PAD may make the presentation of foot infection more subtle than in patients without these problems.

See Online Data Supplement 34 for data supporting Section 7.


**Recommendations for Minimizing Tissue Loss in Patients With PAD**
**COR****LOE****Recommendations**
**I****C-LD**Patients with PAD and diabetes mellitus should be counseled about self–foot examination and healthy foot behaviors.^177,180^
**I****C-LD**In patients with PAD, prompt diagnosis and treatment of foot infection are recommended to avoid amputation.^178,179,181–183^
**IIa****C-LD**In patients with PAD and signs of foot infection, prompt referral to an interdisciplinary care team (Table 8) can be beneficial.^178,184,185^
**IIa****C-EO**It is reasonable to counsel patients with PAD without diabetes mellitus about self–foot examination and healthy foot behaviors.
**IIa****C-EO**Biannual foot examination by a clinician is reasonable for patients with PAD and diabetes mellitus.


## 8. Revascularization For Claudication: Recommendations

A minority of patients with claudication (estimated at <10% to 15% over 5 years or more) will progress to CLI.^186–189^ Therefore, the role of revascularization in claudication is improvement in claudication symptoms and functional status, and consequently in QoL, rather than limb salvage. Revascularization is reasonable when the patient who is being treated with GDMT (including structured exercise therapy) presents with persistent lifestyle-limiting claudication.^13,25,26,190,191^ Lifestyle-limiting claudication is defined by the patient rather than by any test. It includes impairment of activities of daily living and/or vocational and/or recreational activities due to claudication. An individualized approach to revascularization for claudication is recommended for each patient to optimize outcome. Revascularization is but one component of care for the patient with claudication, inasmuch as each patient should have a customized care plan that also includes medical therapy (Section 5), structured exercise therapy (Section 6), and care to minimize tissue loss (Section 7). If a strategy of revascularization for claudication is undertaken, the revascularization strategy should be evidence based and can include endovascular revascularization, surgery, or both.

Due to the variability of ischemic limb symptoms and impact of these symptoms on functional status and QoL, patients should be selected for revascularization on the basis of severity of their symptoms. Factors to consider include a significant disability as assessed by the patient, adequacy of response to medical and structured exercise therapy, status of comorbid conditions, and a favorable risk–benefit ratio. Patient preferences and goals of care are important considerations in the evaluation for revascularization. The revascularization strategy should have a reasonable likelihood of providing durable relief of symptoms. There should be clear discussion with the patient about expected risks and benefits of revascularization, as well as discussion of the durability of proposed procedures. A general recommendation for revascularization as a treatment option for claudication is provided below followed by specific recommendations for endovascular (Section 8.1.1) and surgical (Section 8.1.2) procedures if a revascularization strategy is undertaken.

See Online Data Supplements 35 to 38 for data supporting Section 8.

### 8.1. Revascularization for Claudication


**Recommendation for Revascularization for Claudication**
**COR****LOE****Recommendation**
**IIa****A**Revascularization is a reasonable treatment option for the patient with lifestyle-limiting claudication with an inadequate response to GDMT.^13,25,26,190,191^


#### 8.1.1. Endovascular Revascularization for Claudication

Endovascular techniques to treat claudication include balloon dilation (angioplasty), stents, and atherectomy. These techniques continue to involve and now include covered stents, drug-eluting stents, cutting balloons, and drug-coated balloons. The technique chosen for endovascular treatment is related to lesion characteristics (eg, anatomic location, lesion length, degree of calcification) and operator experience. Assessment of the appropriateness of specific endovascular techniques for specific lesions for the treatment of claudication is beyond the scope of this document.

Revascularization is performed on lesions that are deemed to be hemodynamically significant, and stenoses selected for endovascular treatment should have a reasonable likelihood of limiting perfusion to the distal limb. Stenoses of 50% to 75% diameter by angiography may not be hemodynamically significant, and resting or provoked intravascular pressure measurements may be used to determine whether lesions are significant.^192,193^ Multiple RCTs have compared endovascular procedures to various combinations of medical treatment with or without supervised or unsupervised exercise programs.^13,25,26,190,191,194–206^ These trials have used different endpoints and enrolled patients with anatomic disease distribution at different levels. Long-term patency is greater in the aortoiliac than in the femoropopliteal segment. Furthermore, for femoropopliteal disease, durability is diminished with greater lesion length, occlusion rather than stenosis, the presence of multiple and diffuse lesions, poor-quality runoff, diabetes mellitus, chronic kidney disease, renal failure, and smoking.^207–210^


**Recommendations for Endovascular Revascularization for Claudication**
**COR****LOE****Recommendations**
**I****A**Endovascular procedures are effective as a revascularization option for patients with lifestyle-limiting claudication and hemodynamically significant aortoiliac occlusive disease.^13,25,26,190,194,196,201^
**IIa****B-R**Endovascular procedures are reasonable as a revascularization option for patients with lifestyle-limiting claudication and hemodynamically significant femoropopliteal disease.^190,197–200,205,206^
**IIb****C-LD**The usefulness of endovascular procedures as a revascularization option for patients with claudication due to isolated infrapopliteal artery disease is unknown.^211–213^
**III: Harm****B-NR**Endovascular procedures should not be performed in patients with PAD solely to prevent progression to CLI.^186–189,214–216^


#### 8.1.2. Surgical Revascularization for Claudication

Systematic reviews have concluded that surgical procedures are an effective treatment for claudication and have a positive impact on QoL and walking parameters but have identified sparse evidence supporting the effectiveness of surgery compared with other treatments.^12,191,217,218^ Although symptom and patency outcomes for surgical interventions may be superior to those for less invasive endovascular treatments, surgical interventions are also associated with greater risk of adverse perioperative events^219–225^ Treatment selection should therefore be individualized on the basis of the patient's goals, perioperative risk, and anticipated benefit. Surgical procedures for claudication are usually reserved for individuals who a) do not derive adequate benefit from nonsurgical therapy, b) have arterial anatomy favorable to obtaining a durable result with surgery, and c) have acceptable risk of perioperative adverse events. Acceptable risk is defined by the individual patient and provider on the basis of symptom severity, comorbid conditions, and appropriate GDMT risk evaluation.

The superficial femoral and proximal popliteal arteries are the most common anatomic sites of stenosis or occlusion among individuals with claudication. Femoral-popliteal bypass is therefore one of the most common surgical procedures for claudication. The type of conduit and site of popliteal artery anastomosis (above versus below knee) are major determinants of outcomes associated with femoral-popliteal bypass. Systematic reviews and meta-analyses have identified a clear and consistent primary patency benefit for autogenous vein versus prosthetic grafts for popliteal artery bypass.^226,227^


**Recommendations for Surgical Revascularization for Claudication**
**COR****LOE****Recommendations**
**I****A**When surgical revascularization is performed, bypass to the popliteal artery with autogenous vein is recommended in preference to prosthetic graft material.^226–234^
**IIa****B-NR**Surgical procedures are reasonable as a revascularization option for patients with lifestyle-limiting claudication with inadequate response to GDMT, acceptable perioperative risk, and technical factors suggesting advantages over endovascular procedures.^190,230,235–237^
**III: Harm****B-R**Femoral-tibial artery bypasses with prosthetic graft material should not be used for the treatment of claudication.^238–240^
**III: Harm****B-NR**Surgical procedures should not be performed in patients with PAD solely to prevent progression to CLI.^186–189,241^


## 9. Management Of Cli: Recommendations

Patients with CLI are at increased risk of amputation and major cardiovascular ischemic events. Care of the patient with CLI includes evaluation for revascularization and wound healing therapies, with the objective to minimize tissue loss, completely heal wounds, and preserve a functional foot. Medical therapy to prevent cardiovascular ischemic events is also an important component of care for the patient with CLI (Section 5).

See Online Data Supplements 39 and 40 for data supporting Section 9.

### 9.1. Revascularization for CLI

The goal of surgical or endovascular revascularization in CLI is to provide in-line blood flow to the foot through at least 1 patent artery, which will help decrease ischemic pain and allow healing of any wounds, while preserving a functional limb. The BASIL (Bypass versus Angioplasty in Severe Ischemia of the Leg) RCT^242,243^ demonstrated that endovascular revascularization is an effective option for patients with CLI as compared with open surgery. The primary endpoint of amputation-free survival was the same in the endovascular and surgical arms. Of note, the endovascular arm used only percutaneous transluminal angioplasty.^242,243^ Multiple RCTs comparing contemporary surgical and endovascular treatment for patients with CLI are ongoing.^16–18^ Table 9 addresses factors that may prompt an endovascular versus surgical approach to the patient with CLI.

The angiosome concept has been described in the literature and entails establishing direct blood flow to the infrapopliteal artery directly responsible for perfusing the region of the leg or foot with the nonhealing wound. Multiple retrospective studies and 1 small nonrandomized prospective study assessing the efficacy of this concept have been published.^245–257^ Meta-analyses of these studies found improved wound healing and limb salvage with angiosome-guided therapy but cautioned that the quality of the evidence was low.^258,259^ Although the angiosome concept is theoretically satisfying, randomized data comparing the establishment of in-line flow versus angiosome-guided therapy have yet to be published. Furthermore, there is no evidence yet to demonstrate the potential benefit of treating additional infrapopliteal arteries once in-line flow has been established in one artery, regardless of angiosome.


**Recommendation for Revascularizations for CLI**
**COR****LOE****Recommendation**
**I****B-NR**In patients with CLI, revascularization should be performed when possible to minimize tissue loss.^260^
**I****C-EO**An evaluation for revascularization options should be performed by an interdisciplinary care team (Table 8) before amputation in the patient with CLI.


#### 9.1.1. Endovascular Revascularization for CLI


**Recommendations for Endovascular Revascularization for CLI**
**COR****LOE****Recommendations**
**I****B-R**Endovascular procedures are recommended to establish in-line blood flow to the foot in patients with nonhealing wounds or gangrene.^242,243^
**IIa****C-LD**A staged approach to endovascular procedures is reasonable in patients with ischemic rest pain.^261,262^
**IIa****B-R**Evaluation of lesion characteristics can be useful in selecting the endovascular approach for CLI.^263,264^
**IIb****B-NR**Use of angiosome-directed endovascular therapy may be reasonable for patients with CLI and nonhealing wounds or gangrene.^245,247–249,251–253,255–257^


#### 9.1.2. Surgical Revascularization for CLI


**Recommendations for Surgical Revascularization for CLI**
**COR****LOE****Recommendations**
**I****A**When surgery is performed for CLI, bypass to the popliteal or infrapopliteal arteries (ie, tibial, pedal) should be constructed with suitable autogenous vein.^228,231,234,265^
**I****C-LD**Surgical procedures are recommended to establish in-line blood flow to the foot in patients with nonhealing wounds or gangrene.^266–268^
**IIa****B-NR**In patients with CLI for whom endovascular revascularization has failed and a suitable autogenous vein is not available, prosthetic material can be effective for bypass to the below-knee popliteal and tibial arteries.^269–271^
**IIa****C-LD**A staged approach to surgical procedures is reasonable in patients with ischemic rest pain.^272–274^


### 9.2. Wound Healing Therapies for CLI

A comprehensive plan for treatment of CLI includes a plan to achieve an intact skin surface on a functional foot. The management of patients with CLI and nonhealing wounds includes coordinated efforts for both revascularization and wound healing among members of an interdisciplinary care team (Table 8). The structure and activities of interdisciplinary care teams for CLI may vary according to several factors, including the local availability of resources. Revascularization is coordinated with the efforts of clinicians who manage foot infections, provide offloading, and achieve complete wound healing, either through medical therapy, surgical options, or a combination of these options.

See Online Data Supplement 34a for a complete list of functions of the interdisciplinary care team.


**Recommendations for Wound Healing Therapies for CLI**
**COR****LOE****Recommendations**
**I****B-NR**An interdisciplinary care team should evaluate and provide comprehensive care for patients with CLI and tissue loss to achieve complete wound healing and a functional foot.^184,275–277^
**I****C-LD**In patients with CLI, wound care after revascularization should be performed with the goal of complete wound healing.^275^
**IIb****B-NR**In patients with CLI, intermittent pneumatic compression (arterial pump) devices may be considered to augment wound healing and/or ameliorate severe ischemic rest pain.^278^
**IIb****C-LD**In patients with CLI, the effectiveness of hyperbaric oxygen therapy for wound healing is unknown.^279^
**III: No Benefit****B-R**Prostanoids are not indicated in patients with CLI.^280^


## 10. Management Of Acute Limb Ischemia: Recommendations

Acute limb ischemia (ALI) is one of the most treatable and potentially devastating presentations of PAD. Timely recognition of arterial occlusion as the cause of an ischemic, cold, painful leg is crucial to successful treatment. The writing committee has used a standard definition of ALI in which symptom duration is <2 weeks (Table 2).^21,22^ Category I refers to viable limbs that are not immediately threatened. Category II refers to threatened limbs. Category IIa limbs are marginally threatened and salvageable, if promptly treated. Category IIb are immediately threatened limbs that require immediate revascularization if salvage is to be accomplished. Category III are irreversibly damaged limbs, in which case resultant major tissue loss or permanent nerve damage is inevitable.^22^

Patients with ALI should be rapidly evaluated by a vascular specialist if one is available. Depending on local clinical expertise, the vascular specialist may be a vascular surgeon, interventional radiologist, cardiologist, or a general surgeon with specialized training and experience in treating PAD. If such expertise is not locally or rapidly available, there should be strong consideration of transfer of the patient to a facility with such resources. The more advanced the degree of ischemia, the more rapidly the communication (eg, with regard to potential patient transfer) needs to occur.

**Figure 3:**
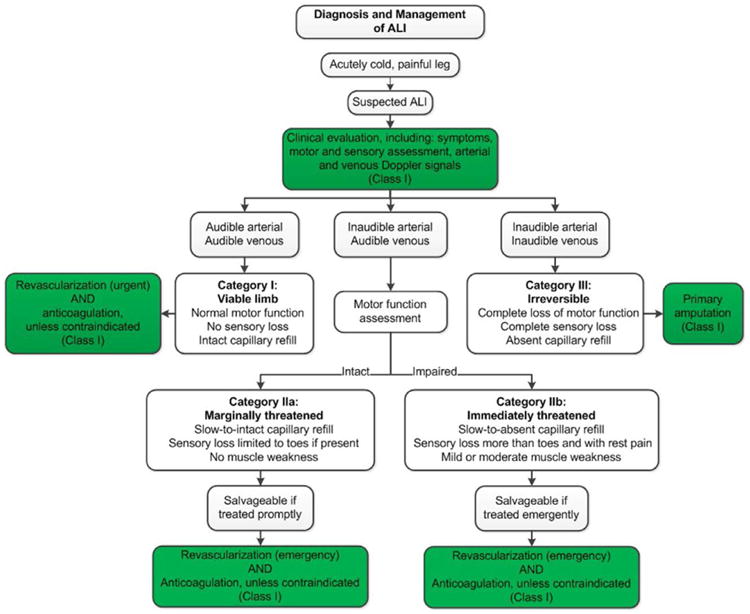
Diagnosis and Management of ALI ^21,22^ Colors correspond to Class of Recommendation in Table 1. ALI indicates acute limb ischemia.

ALI is a medical emergency and must be recognized rapidly. The time constraint is due to the period that skeletal muscle will tolerate ischemia—roughly 4 to 6 hours.^281^ A rapid assessment of limb viability and ability to restore arterial blood flow should be performed by a clinician able to either complete the revascularization or triage the patient.^282^ Lower extremity symptoms in ALI can include both pain and loss of function. The longer these symptoms are present, the less likely the possibility of limb salvage.^283,284^ Clinical assessment must include symptom duration, pain intensity, and motor and sensory deficit severity to distinguish a threatened from a nonviable extremity (Figure 3). The bedside assessment includes arterial and venous examination with a handheld continuous-wave Doppler because of the inaccuracy of pulse palpation.^22^ The loss of Dopplerable arterial signal indicates that the limb is threatened. The absence of both arterial and venous Doppler signal indicates that the limb may be irreversibly damaged (nonsalvageable). Comorbidities should be investigated and managed aggressively, but this must not delay therapy. Even in the setting of rapid and effective revascularization, the 1-year morbidity and mortality rates ALI are high.^283,285^

See Figure 3 for the algorithm on diagnosis and management of ALI and Online Data Supplements 45 to 50 for data supporting Section 10.

### 10.1. Clinical Presentation of ALI


**Recommendations for Clinical Presentation of ALI**
**COR****LOE****Recommendations**
**I****C-EO**Patients with ALI should be emergently evaluated by a clinician with sufficient experience to assess limb viability and implement appropriate therapy.
**I****C-LD**In patients with suspected ALI, initial clinical evaluation should rapidly assess limb viability and potential for salvage and does not require imaging.^282–284,286,287^


### 10.2. Medical Therapy for ALI


**Recommendation for ALI Medical Therapy**
**COR****LOE****Recommendation**
**I****C-EO**In patients with ALI, systemic anticoagulation with heparin should be administered unless contraindicated.


### 10.3. Revascularization for ALI

For marginally or immediately threatened limbs (Category IIa and IIb ALI), revascularization should be performed emergently (within 6 hours). For viable limbs (Category I ALI), revascularization should be performed an on urgent basis (within 6–24 hours). The revascularization strategy can range from catheter-directed thrombolysis to surgical thromboembolectomy. Available facilities and clinical expertise are factors that should be considered when determining the revascularization strategy. The technique that will provide the most rapid restoration of arterial flow with the least risk to the patient should be selected. For example, catheter-directed thrombolysis can provide rapid restoration of arterial flow to a viable or marginally threatened limb, particularly in the setting of recent occlusion, thrombosis of synthetic grafts, and stent thrombosis.^288^ If this is not available locally, surgical options for timely revascularization should be considered, along with the feasibility of timely transfer to a facility with the necessary expertise.

Prolonged duration of ischemia is the most common factor in patients requiring amputation for treatment of ALI. The risks associated with reconstruction outweigh the potential benefit in a limb that is already insensate or immobile because of prolonged ischemia. Patients who have an insensate and immobile limb in the setting of prolonged ischemia (>6 to 8 hours) are unlikely to have potential for limb salvage with revascularization.


**Recommendations for Revascularization for ALI**
**COR****LOE****Recommendations**
**I****C-LD**In patients with ALI, the revascularization strategy should be determined by local resources and patient factors (eg, etiology and degree of ischemia).^288–290^
**I****A**Catheter-based thrombolysis is effective for patients with ALI and a salvageable limb.^288–292^
**I****C-LD**Amputation should be performed as the first procedure in patients with a nonsalvageable limb.^293,294^
**I****C-LD**Patients with ALI should be monitored and treated (eg, fasciotomy) for compartment syndrome after revascularization.^293,294^
**IIa****B-NR**In patients with ALI with a salvageable limb, percutaneous mechanical thrombectomy can be useful as adjunctive therapy to thrombolysis.^295–299^
**IIa****C-LD**In patients with ALI due to embolism and with a salvageable limb, surgical thromboembolectomy can be effective.^300–302^
**IIb****C-LD**The usefulness of ultrasound-accelerated catheter-based thrombolysis for patients with ALI with a salvageable limb is unknown.^303–305^


### 10.4. Diagnostic Evaluation of the Cause of ALI

ALI may be related to underlying PAD (including prior lower extremity bypass graft) or may be related to other conditions that can result in ALI through either thrombotic (eg, hypercoagulable state) or embolic mechanisms. Treatment of ALI should not be delayed for testing for the underlying cause of the limb ischemia because delay from symptom onset to revascularization is a major determinant of outcome.^283,284^ The evaluation of a cardiovascular (ie, embolic) cause for ALI is most useful in the patient without underlying PAD and can be completed after revascularization. Evaluation for cardiovascular cause includes electrocardiogram or additional heart rhythm monitoring to detect atrial fibrillation, electrocardiogram to detect evidence of MI, and echocardiography to further determine whether there is a cardiac etiology for thromboembolism, such as valvular vegetation, left atrial or left ventricular thrombus, or intracardiac shunt.


**Recommendations for Diagnostic Evaluation of the Cause of ALI**
**COR****LOE****Recommendations**
**I****C-EO**In the patient with ALI, a comprehensive history should be obtained to determine the cause of thrombosis and/or embolization.
**IIa****C-EO**In the patient with a history of ALI, testing for a cardiovascular cause of thromboembolism can be useful.


## 11. Longitudinal Follow-Up: Recommendations

PAD is a lifelong chronic medical condition. A comprehensive care plan for patients with PAD includes periodic clinical evaluation by a healthcare provider with experience in the care of vascular patients. Ongoing care focuses on cardiovascular risk reduction with medical therapy, optimizing functional status with structured exercise, and, when indicated, revascularization. The care plan is further customized depending on whether the patient has undergone a revascularization procedure.

See Online Data Supplements 51 and 52 for data supporting Section 11.


**Recommendations for Longitudinal Follow-Up**
**COR****LOE****Recommendations**
**I****C-EO**Patients with PAD should be followed up with periodic clinical evaluation, including assessment of cardiovascular risk factors, limb symptoms, and functional status.
**I****C-EO**Patients with PAD who have undergone lower extremity revascularization (surgical and/or endovascular) should be followed up with periodic clinical evaluation and ABI measurement.
**IIa****B-R**Duplex ultrasound can be beneficial for routine surveillance of infrainguinal, autogenous vein bypass grafts in patients with PAD.^306–312^
**IIa****C-LD**Duplex ultrasound is reasonable for routine surveillance after endovascular procedures in patients with PAD.^313–315^
**IIb****B-R**The effectiveness of duplex ultrasound for routine surveillance of infrainguinal prosthetic bypass grafts in patients with PAD is uncertain.^310,316–318^


## 12. Evidence Gaps and Future Research Directions

In performing the evidence review and in developing the present guidelines, the writing committee identified the following critical evidence gaps and future directions for PAD-related research:

- Basic science and translational studies to better understand the vascular biology of endovascular therapies and bypass grafting and to develop new methods for preventing restenosis after revascularization.
- Determination of risk factors for progression from asymptomatic PAD to symptomatic disease, including CLI.
- RCTs needed to determine the value of using the ABI to identify asymptomatic patients with PAD for therapies to reduce cardiovascular risk (eg, antiplatelet agents, statins, and other therapies).
- Advancement in PAD diagnostics, such as technologies for simplified yet highly accurate measurement of the ABI and tools for more reliable noninvasive perfusion assessment in CLI.
- Comparative-effectiveness studies to determine the optimal antiplatelet therapy (drug or drugs and dosage) for prevention of cardiovascular and limb-related events in patients with PAD.
- Development of additional medical therapies for claudication—an area of unmet medical need with a currently limited research pipeline.^319^
- Studies to investigate the role of dietary intervention, in addition to statin therapy, to improve outcome and modify the natural history of PAD.
- Additional research to identify the best community-or home-based exercise programs for patients with PAD to maximize functional status and improve QoL, as well as the role of such exercise programs before or in addition to revascularization.
- Development and validation of improved clinical classification systems for PAD that incorporate symptoms, anatomic factors, and patient-specific risk factors and can be used to predict clinical outcome and optimize treatment approach. An example of a recently developed classification system is the Society for Vascular Surgery limb classification system, based on wound, ischemia, and foot infection (WIfI), which has been validated in different populations and may permit more meaningful prognosis in patients with CLI.^320–324^
- Comparative- and cost-effectiveness studies of the different endovascular technologies for treatment of claudication and CLI, including drug-coated balloons and drug-eluting stents. Studies should include patient-centered endpoints, such as functional parameters, time to wound healing, and QoL, in addition to standard patency-focused outcomes. These studies could then be incorporated into value-based clinical algorithms for approach to revascularization for claudication and CLI.
- Additional studies to demonstrate the impact of multisocietal registries on clinical outcomes and appropriate use. At present, these include: the Vascular Quality Initiative (VQI), the National Cardiovascular Data Registry Peripheral Vascular Intervention Registry™ (PVI Registry™), and the National Radiology Data Registry for Interventional Radiology (NRDR). These registries provide an opportunity to obtain “real-world” data on surgical and endovascular procedures for PAD and improve quality by providing feedback to participating centers. Future efforts should incorporate these registries into interventional RCTs and post-marketing studies of PAD-related devices.

## 13. Advocacy Priorities

The writing committee identified 3 priorities for multisocietal advocacy initiatives to improve health care for patients with PAD. First, the writing committee supports the availability of the ABI as the initial diagnostic test to establish the diagnosis of PAD in patients with history or physical examination findings suggestive of PAD (Table 4). Although the ABI test is generally reimbursed by third-party payers for patients with classical claudication or lower extremity wounds, payers may not provide reimbursement for the ABI with other findings suggestive of PAD, such as lower extremity pulse abnormalities or femoral bruits. The writing committee affirms the importance of confirming the diagnosis of PAD in such patients to allow for GDMT as delineated in this document. Second, the writing committee supports the vital importance of insuring access to supervised exercise programs for patients with PAD. Although extensive high-quality evidence supports supervised exercise programs to improve functional status and QoL, only a minority of patients with PAD participate in such programs because of lack of reimbursement by third-party payers. Third, the writing committee recognizes the need for incorporation of patient-centered outcomes into the process of regulatory approval of new medical therapies and revascularization technologies. For revascularization technologies, regulatory approval is driven primarily by data on angiographic efficacy (ie, target-lesion patency) and safety endpoints. The nature of the functional limitation associated with PAD warrants the incorporation of patient-centered outcomes, such as functional parameters and QoL, into the efficacy outcomes for the approval process.

## Supplementary Material

Evidence Table 1. Nonrandomized Trials, Observational Studies, and/or Registries of History for Clinical Assessment for PAD–Section 2.1.
Evidence Table 2. Nonrandomized Trials, Observational Studies, and/or Registries of Physical Examination for Clinical Assessment for PAD–Section 2.1.
Evidence Table 3. RCTs of Resting ABI for Diagnosing PAD–Section 3.1.
Evidence Table 4. Nonrandomized Trials, Observational Studies, and/or Registries of Resting ABI for Diagnosing PAD–Section 3.1.
Evidence Table 5. Nonrandomized Trials, Observational Studies, and/or Registries of Physiological Testing–Section 3.2.
Evidence Table 6. Nonrandomized Trials, Observational Studies, and/or Registries of Imaging for Anatomic Assessment (Ultrasound, CTA, MRA, Angiography)–Section 3.3.
Evidence Table 7. RCTs of Imaging for Anatomic Assessment (Ultrasound, CTA, MRA, Angiography)–Section 3.3.
Evidence Table 8. Nonrandomized Trials, Observational Studies, and/or Registries for Abdominal Aortic Aneurysm–Section 4.1.
Evidence Table 9. Nonrandomized Trials, Observational Studies, and/or Registries of Coronary Artery Disease Screening in PAD–Section 4.2.
Evidence Table 10. RCTs for CAD Screening in PAD–Section 4.2.
Evidence Table 11. Nonrandomized Trials, Observational Studies, and/or Registries of Screening in Carotid Artery Disease–Section 4.3.
Evidence Table 12. Nonrandomized Trials, Observational Studies, and/or Registries for Renal Artery Disease–Section 4.4.
Evidence Table 13. RCTs Evaluating Antiplatelet Agents– Section 5.1.
Evidence Table 14. Nonrandomized Trials, Observational Studies, and/or Registries of Antiplatelet Agents–Section 5.2.
Evidence Table 15. Randomized Trials Comparing Statin Agents–Section 5.2.
Evidence Table 16. Nonrandomized Trials, Observational Studies, and/or Registries of Statin Agents–Section 5.2.
Evidence Table 17. RCTs for Antihypertensive Agents– Section 5.3.
Evidence Table 18. Nonrandomized Trials, Observational Studies, and/or Registries of Antihypertensive Agents–Section 5.3.
Evidence Table 19. RCTs for Smoking Cessation–Section 5.4.
Evidence Table 20. Nonrandomized Trials, Observational Studies, and/or Registries of Smoking Cessation–Section 5.4.
Evidence Table 21. RCTs Evaluating Glycemic Control in Patients with PAD and Diabetes Mellitus–Section 5.5.
Evidence Table 22. Nonrandomized Trials, Observational Studies, and/or Registries of Glycemic Control–Section 5.5.
Evidence Table 23. RCTs Evaluating Oral Anticoagulation–Section 5.6.
Evidence Table 24. Nonrandomized Trials, Observational Studies, and/or Registries of Oral Anticoagulation–Section 5.6.
Evidence Table 25. RCTs and Observational Studies of Cilostazol–Section 5.7.
Evidence Table 26. Nonrandomized Trials, Observational Studies, and/or Registries of Pentoxifylline–Section 5.8.
Evidence Table 27. Systematic Review of Chelation Therapy–Section 5.9.
Evidence Table 28. Nonrandomized Trials, Observational Studies, and/or Registries of Homocysteine Lowering Therapy for Lower Extremity PAD in Patients with Diabetes Mellitus–Section 5.10.1.
Evidence Table 29. RCTs Comparing Additional Medical Therapies of Homocysteine Lowering Therapy for Lower Extremity PAD–Section 5.10.1.
Evidence Table 30. RCTs for Influenza Vaccination–Section 5.10.2.
Evidence Table 31. Nonrandomized Trials for Influenza Vaccination–Section 5.10.2.
Evidence Table 32. RCTs for Exercise Therapy–Section 6.
Evidence Table 33. Nonrandomized Trials, Observational Studies, and/or Registries for Exercise Therapy–Section 6.
Evidence Table 34. Nonrandomized Trials and Observational Studies of Minimizing Tissue Loss in Patients with PAD–Section 7.
Data Supplement 34a. Functions of a Multidisciplinary Foot Care / Amputation Prevention Team–Section 7.
Evidence Table 35. RCTs Comparing Endovascular Treatment and Endovascular Versus Noninvasive Treatment of Claudication–Section 8.1.
Evidence Table 36. Nonrandomized Trials, Observational Studies, and/or Registries of Endovascular and Endovascular Versus Noninvasive Treatment of Claudication–Section 8.1.
Evidence Table 37. RCTs Evaluating Surgical Treatment for Claudication–Section 8.1.2.
Evidence Table 38. Nonrandomized Trials, Observational Studies, and/or Registries of Surgical Treatment for Claudication–Section 8.1.2.
Evidence Table 39. RCTs Comparing Endovascular Revascularization for Chronic CLI–Section 8.2.
Evidence Table 40. Nonrandomized Trials, Observational Studies, and/or Registries of Endovascular Revascularization for Chromic CLI–Section 8.2.1.
Evidence Table 41. RCTs of Surgical Revascularization for Chronic CLI–Section 8.2.
Evidence Table 42. Nonrandomized Trials, Observational Studies, and/or Registries for Surgical Revascularization for Chronic CLI–Section 8.2.
Evidence Table 43. RCT Comparing Prostanoids for End-Stage Peripheral Artery Disease–Section 8.2.3.
Evidence Table 44. Nonrandomized Trials, Observational Studies, and/or Registries for Would Healing Therapies for CLI–Section 8.2.3.
Evidence Table 45. Nonrandomized Trials, Observational Studies, and/or Registries of Acute Limb Ischemia–Section 9.1.
Evidence Table 46. Nonrandomized Trials, Observational studies, and/or Registries Comparing Evaluating Noninvasive Testing and Angiography for ALI–Section 9.1.
Evidence Table 47. RCTs of Revascularization Strategy for ALI–Section 9.2.2.
Evidence Table 48. Nonrandomized Trials, Observational Studies, and/or Registries of Clinical Presentation of ALI–Section 9.2.2.
Evidence Table 49. Nonrandomized Trials, Observational Studies, and/or Registries of Diagnostic Evaluation of the Cause of ALI–Section 9.2.2.
Evidence Table 50. Nonrandomized Trials, Observational Studies, and/or Registries of Revascularization Strategy for ALI–Section 9.2.2.
Evidence Table 51. RCTs for Longitudinal Follow-Up–Section 10.
Evidence Table 52. Nonrandomized Trials, Observational Studies, and/or Registries for Longitudinal Follow-Up–Section 10.

## Figures and Tables

**Table 1:** ACC/AHA Recommendation System: Applying Class of Recommendation and Level of Evidence to Clinical Strategies, Interventions, Treatments, or Diagnostic Testing in Patient Care* (Updated August 2015)

CLASS (STRENGTH) OF RECOMMENDATION	
**Class I (strong)**	**Benefit >>> Risk**
Suggested phrases for writing recommendations: ■ Is recommended ■ Is indicated/useful/effective/beneficial ■ Should be performed/administered/other ■ Comparative-Effectiveness Phrases†: ○ Treatment/strategy A is recommended/indicated in preference to treatment B ○ Treatment A should be chosen over treatment B	
**CLASS IIa (MODERATE)**	**Benefit >> Risk**
Suggested phrases for writing recommendations; ■ Is reasonable ■ Can be useful/effective/beneficial ■ Comparative-Effectiveness Phrases†: ○ Treatment/strategy A is probably recommended/indicated in preference to treatment B ○ It is reasonable to choose treatment A over treatment B	
**CLASS IIb (WEAK)**	**Benefit ≥ Risk**
Suggested phrases for writing recommendations: ■ May/might be reasonable ■ May/might be considered ■ Usefulness/effectiveness is unknown/unclear/uncertain or not well established	
**CLASS III: No Benefit (MODERATE) (*Generally, LOE A or B use only*)**	**Benefit = Risk**
Suggested phrases for writing recommendations: ■ Is not recommended ■ Is not indicated/useful/effective/beneficial ■ Should not be performed/administered/other	
**CLASS III: Harm (STRONG)**	**Risk > Benefit**
Suggested phrases for writing recommendations: ■ Potentially harmful ■ Causes harm ■ Associated with excess morbidity/mortality ■ Should not be performed/administered/other	
**LEVEL (QUALITY) OF EVIDENCE**‡	
**LEVEL A**	
■ High-quality evidence‡ from more than 1 RCT ■ Meta-analyses of high-quality RCTs ■ One or more RCTs corroborated by high-quality registry studies	
**LEVEL B-R**	**(Randomized)**
■ Moderate-quality evidence‡ from 1 or more RCTs ■ Meta-analyses of moderate-quality RCTs	
**LEVEL B-NR**	**(Nonrandomized)**
■ Moderate-quality evidence‡ from 1 or more well-designed, well-executed nonrandomized studies, observational studies, or registry studies ■ Meta-analyses of such studies	
**LEVEL C-LD**	**(Limited Data)**
■ Randomized or nonrandomized observational or registry studies with limitations of design or execution ■ Meta-analyses of such studies ■ Physiological or mechanistic studies in human subjects	
**LEVEL C-E0**	**(Expert Opinion)**
Consensus of expert opinion based on clinical experience	

COR and LOE are determined independently (any COR may be paired with any LOE). A recommendation with LOE C does not imply that the recommendation is weak. Many important clinical questions addressed in guidelines do not lend themselves to clinical trials. Although RCTs are unavailable, there may be a very dear clinical consensus that a particular test or therapy is useful or effective.

* The outcome or result of the intervention should be specified (an improved clinical outcome or increased diagnostic accuracy or incremental prognostic information).

† For comparative-effectiveness recommendations (COR 1 and lla; LOE A and B only), studies that support the use of comparator verbs should involve direct comparisons of the treatments or strategies being evaluated.

‡ The method of assessing quality is evolving, including the application of standardized widely used, and preferably validated evidence grading tools; and for systematic reviews, the incorporation of an Evidence Review Committee.

COR indicates Class of Recommendation; EO, expert opinion; LD, limited data; LOE, Level of Evidence; NR, nonrandomized; R, randomized; and RCT randomized controlled trial.

**Table 2:** Definition of PAD Key Terms

Term	Definition
Claudication	Fatigue, discomfort, cramping, or pain of vascular origin in the muscles of the lower extremities that is consistently induced by exercise and consistently relieved by rest (within 10 min).
Acute limb ischemia (ALI)	Acute (<2 wk), severe hypoperfusion of the limb characterized by these features: pain, pallor, pulselessness, poikilothermia (cold), paresthesias, and paralysis. One of these categories of ALI is assigned (Section 10): Viable—Limb is not immediately threatened; no sensory loss; no muscle weakness; audible arterial and venous Doppler. Threatened—Mild-to-moderate sensory or motor loss; inaudible arterial Doppler; audible venous Doppler; may be further divided into IIa (marginally threatened) or IIb (immediately threatened). Irreversible—Major tissue loss or permanent nerve damage inevitable; profound sensory loss, anesthetic; profound muscle weakness or paralysis (rigor); inaudible arterial and venous Doppler.^21,22^
Tissue loss	Type of tissue loss: Minor—nonhealing ulcer, focal gangrene with diffuse pedal ischemia. Major—extending above transmetatarsal level; functional foot no longer salvageable.^21^
Critical limb ischemia (CLI)	A condition characterized by chronic (≥2 wk) ischemic rest pain, nonhealing wound/ulcers, or gangrene in 1 or both legs attributable to objectively proven arterial occlusive disease. The diagnosis of CLI is a constellation of both symptoms and signs. Arterial disease can be proved objectively with ABI, TBI, TcPO_2_, or skin perfusion pressure. Supplementary parameters, such as absolute ankle and toe pressures and pulse volume recordings, may also be used to assess for significant arterial occlusive disease. However, a very low ABI or TBI does not necessarily mean the patient has CLI. The term CLI implies chronicity and is to be distinguished from ALI.^23^
In-line blood flow	Direct arterial flow to the foot, excluding collaterals.
Functional status	Patient's ability to perform normal daily activities required to meet basic needs, fulfill usual roles, and maintain health and well-being. Walking ability is a component of functional status.
Nonviable limb	Condition of extremity (or portion of extremity) in which loss of motor function, neurological function, and tissue integrity cannot be restored with treatment.
Salvageable limb	Condition of extremity with potential to secure viability and preserve motor function to the weight-bearing portion of the foot if treated.
Structured exercise program	Planned program that provides individualized recommendations for type, frequency, intensity, and duration of exercise. Program provides recommendations for exercise progression to assure that the body is consistently challenged to increase exercise intensity and levels as functional status improves over time. There are 2 types of structured exercise program for patients with PAD: Supervised exercise program Structured community- or home-based exercise program
Supervised exercise program	Structured exercise program that takes place in a hospital or outpatient facility in which intermittent walking exercise is used as the treatment modality. Program can be standalone or can be made available within a cardiac rehabilitation program. Program is directly supervised by qualified healthcare provider(s). Training is performed for a minimum of 30 to 45 min per session, in sessions performed at least 3 times/wk for a minimum of 12 wk.^24–34^ Patients may not initially achieve these targets, and a treatment goal is to progress to these levels over time. Training involves intermittent bouts of walking to moderate-to-maximum claudication, alternating with periods of rest. Warm-up and cool-down periods precede and follow each session of walking.
Structured community- or home-based exercise program	Structured exercise program that takes place in the personal setting of the patient rather than in a clinical setting.^29,35–39^ Program is self-directed with the guidance of healthcare providers who prescribe an exercise regimen similar to that of a supervised program. Patient counseling ensures that patients understand how to begin the program, how to maintain the program, and how to progress the difficulty of the walking (by increasing distance or speed). Program may incorporate behavioral change techniques, such as health coaching and/or use of activity monitors.
Emergency versus urgent	An *emergency* procedure is one in which life or limb is threatened if the patient is not in the operating room or interventional suite and/or where there is time for no or very limited clinical evaluation, typically within <6 h. An *urgent* procedure is one in which there may be time for a limited clinical evaluation, usually when life or limb is threatened if the patient is not in the operating room or interventional suite, typically between 6 and 24 h.
Interdisciplinary care team	A team of professionals representing different disciplines to assist in the evaluation and management of the patient with PAD. For the care of patients with CLI, the interdisciplinary care team should include individuals who are skilled in endovascular revascularization, surgical revascularization, wound healing therapies and foot surgery, and medical evaluation and care. Interdisciplinary care team members may include: Vascular medical and surgical specialists (ie, vascular medicine, vascular surgery, interventional radiology, interventional cardiology) Nurses Orthopedic surgeons and podiatrists Endocrinologists Internal medicine specialists Infectious disease specialists Radiology and vascular imaging specialists Physical medicine and rehabilitation clinicians Orthotics and prosthetics specialists Social workers Exercise physiologists Physical and occupational therapists Nutritionists/dieticians
Cardiovascular ischemic events	Acute coronary syndrome (acute MI, unstable angina), stroke, or cardiovascular death.
Limb-related events	Worsening claudication, new CLI, new lower extremity revascularization, or new ischemic amputation.

ABI indicates ankle-brachial index; ALI, acute limb ischemia; CLI, critical limb ischemia; MI, myocardial infarction; PAD, peripheral artery disease; TBI, toe-brachial index; and TcPO_2_, transcutaneous oxygen pressure.

**Table 3:** Patients at Increased Risk of PAD

Age ≥65 y
Age 50–64 y, with risk factors for atherosclerosis (eg, diabetes mellitus, history of smoking, hyperlipidemia, hypertension) or family history of PAD^52^
Age <50 y, with diabetes mellitus and 1 additional risk factor for atherosclerosis
Individuals with known atherosclerotic disease in another vascular bed (eg, coronary, carotid, subclavian, renal, mesenteric artery stenosis, or AAA)

AAA indicates abdominal aortic aneurysm; PAD, peripheral artery disease.

**Table 4:** History and/or Physical Examination Findings Suggestive of PAD

History
Claudication
Other non–joint-related exertional lower extremity symptoms (not typical of claudication)
Impaired walking function
Ischemic rest pain
Physical Examination
Abnormal lower extremity pulse examination
Vascular bruit
Nonhealing lower extremity wound
Lower extremity gangrene
Other suggestive lower extremity physical findings (eg, elevation pallor/dependent rubor)

PAD indicates peripheral artery disease.

**Table 5:** Alternative Diagnoses for Leg Pain or Claudication With Normal Physiological Testing (Not PAD-Related)

Condition	Location	Characteristic	Effect of Exercise	Effect of Rest	Effect of Position	Other Characteristics
Symptomatic Baker's cyst	Behind knee, down calf	Swelling, tenderness	With exercise	Also present at rest	None	Not intermittent
Venous claudication	Entire leg, worse in calf	Tight, bursting pain	After walking	Subsides slowly	Relief speeded by elevation	History of iliofemoral deep vein thrombosis; edema; signs of venous stasis
Chronic compartment syndrome	Calf muscles	Tight, bursting pain	After much exercise (jogging)	Subsides very slowly	Relief with rest	Typically heavy muscled athletes
Spinal stenosis	Often bilateral buttocks, posterior leg	Pain and weakness	May mimic claudication	Variable relief but can take a long time to recover	Relief by lumbar spine flexion	Worse with standing and extending spine
Nerve root compression	Radiates down leg	Sharp lancinating pain	Induced by sitting, standing, or walking	Often present at rest	Improved by change in position	History of back problems; worse with sitting; relief when supine or sitting
Hip arthritis	Lateral hip, thigh	Aching discomfort	After variable degree of exercise	Not quickly relieved	Improved when not weight bearing	Symptoms variable; history of degenerative arthritis
Foot/ankle arthritis	Ankle, foot, arch	Aching pain	After variable degree of exercise	Not quickly relieved	May be relieved by not bearing weight	Symptoms variable; may be related to activity level or present at rest

Modified from Norgren L et al.^23^ PAD indicates peripheral artery disease.

**Table 6:** Alternative Diagnoses for Nonhealing Wounds With Normal Physiological Testing (Not PAD-Related)

Condition	Location	Characteristics and Causes
Venous ulcer	Distal leg, especially above medial mellolus	Develops in regions of skin changes due to chronic venous disease and local venous hypertension Typically wet (ie, wound drainage) rather than dry lesion
Distal small arterial occlusion (microangiopathy)	Toes, foot, leg	End-stage renal disease Thromboangiitis obliterans (Buerger's) Sickle-cell anemia Vasculitis (eg, Churg-Strauss, Henoch-Schonlein purpura, leukocytoclastic vasculitis, microscopic polyangiitis, polyarteritis nodosa) Scleroderma Cryoagglutination Embolic (eg, cholesterol emboli, thromboemboli, endocarditis) Thrombotic (eg, antiphospholipid antibody syndrome, Sneddon's syndrome, warfarin skin necrosis, disseminated intravascular coagulation, livedoid vasculitis, protein C or S deficiency, prolonged vasospasm)
Local injury	Toes, foot, leg	Trauma Insect or animal bite Burn
Medication related	Toes, foot, leg	Drug reactions (eg, erythema multiforme) Medication direct toxicity (eg, doxorubicin, hydroxyurea, some tyrosine kinase inhibitors)
Neuropathic	Pressure zones of foot	Hyperkeratosis surrounds the ulcer Diabetes mellitus with peripheral neuropathy Peripheral neuropathy without diabetes mellitus Leprosy
Autoimmune injury	Toes, foot, leg	With blisters (eg, pemphigoid, pemphigus, epidermolysis bullosa) Without blisters (eg, dermatomyositis, lupus, scleroderma)
Infection	Toes, foot, leg	Bacterial (eg, pseudomonas, necrotizing streptococcus) Fungal (eg, blastomycosis, Madura foot, chromomycosis) Mycobacterial Parasitic (eg, Chagas, leishmaniasis) Viral (eg, herpes)
Malignancy	Toes, foot, leg	Primary skin malignancy Metastatic malignancy Malignant transformation of ulcer
Inflammatory	Toes, foot, leg	Necrobiosis lipoidica Pyoderma gangrenosum Granuloma annulare

PAD indicates peripheral artery disease.

**Table 7:** Structured Exercise Programs for PAD: Definitions

Supervised exercise program (COR I, LOE A)
Program takes place in a hospital or outpatient facility.
Program uses intermittent walking exercise as the treatment modality.
Program can be standalone or within a cardiac rehabilitation program.
Program is directly supervised by qualified healthcare provider(s).
Training is performed for a minimum of 30–45 min/session; sessions are performed at least 3 times/wk for a minimum of 12 wk.^24–34^
Training involves intermittent bouts of walking to moderate-to-maximum claudication, alternating with periods of rest.
Warm-up and cool-down periods precede and follow each session of walking.
Structured community- or home-based exercise program (COR IIa, LOE A)
Program takes place in the personal setting of the patient rather than in a clinical setting.^29,35–39^
Program is self-directed with guidance of healthcare providers.
Healthcare providers prescribe an exercise regimen similar to that of a supervised program.
Patient counseling ensures understanding of how to begin and maintain the program and how to progress the difficulty of the walking (by increasing distance or speed).
Program may incorporate behavioral change techniques, such as health coaching or use of activity monitors.

COR indicates Class of Recommendation; LOE, Level of Evidence; and PAD, peripheral artery disease.

**Table 8:** Interdisciplinary Care Team for PAD

A team of professionals representing different disciplines to assist in the evaluation and management of the patient with PAD. For the care of patients with CLI, the interdisciplinary care team should include individuals who are skilled in endovascular revascularization, surgical revascularization, wound healing therapies and foot surgery, and medical evaluation and care.
Interdisciplinary care team members may include:
Vascular medical and surgical specialists (ie, vascular medicine, vascular surgery, interventional radiology, interventional cardiology)
Nurses
Orthopedic surgeons and podiatrists
Endocrinologists
Internal medicine specialists
Infectious disease specialists
Radiology and vascular imaging specialists
Physical medicine and rehabilitation clinicians
Orthotics and prosthetics specialists
Social workers
Exercise physiologists
Physical and occupational therapists
Nutritionists/dieticians

CLI indicates critical limb ischemia; and PAD, peripheral artery disease.

**Table 9:** Therapy for CLI: Findings That Prompt Consideration of Surgical or Endovascular Revascularization

Findings That Favor Consideration of Surgical Revascularization	Examples
Factors associated with technical failure or poor durability with endovascular treatment	Lesion involving common femoral artery, including origin of deep femoral artery
	Long segment lesion involving the below-knee popliteal and/or infrapopliteal arteries in a patient with suitable single-segment autogenous vein conduit
	Diffuse multilevel disease that would require endovascular revascularization at multiple anatomic levels
	Small-diameter target artery proximal to site of stenosis or densely calcified lesion at location of endovascular treatment
Endovascular treatment likely to preclude or complicate subsequent achievement of in-line blood flow through surgical revascularization	Single-vessel runoff distal to ankle
Findings That Favor Consideration of Endovascular Revascularization	Examples
The presence of patient comorbidities may place patients at increased risk of perioperative complications from surgical revascularization. In these patients, an endovascular-first approach should be used regardless of anatomy	Patient comorbidities, including coronary ischemia, cardiomyopathy, congestive heart failure, severe lung disease, and chronic kidney disease
Patients with rest pain and disease at multiple levels may undergo a staged approach as part of endovascular-first approach	In-flow disease can be addressed first, and out-flow disease can be addressed in a staged manner, when required, if clinical factors or patient safety prevent addressing all diseased segments at one setting
Patients without suitable autologous vein for bypass grafts	Some patients have had veins harvested for previous coronary artery bypass surgery and do not have adequate remaining veins for use as conduits. Similarly, patients may not have undergone prior saphenous vein harvest, but available vein is of inadequate diameter

CLI indicates critical limb ischemia.

## Appendix 1

**Table:** Author Relationships With Industry and Other Entities (Relevant)—2016 AHA/ACC Guideline on the Management of Patients With Lower Extremity Peripheral Artery Disease (March 2014)

Committee Member	Employment	Consultant	Speakers Bureau	Ownership/Partnership/Principal	Personal Research	Institutional, Organizational, or Other Financial Benefit	Expert Witness	Voting Recusals by Section*
Marie D. Gerhard-Herman, Chair	Harvard Medical School—Associate Professor	None	None	None	None	None	None	None
Heather L. Gornik, Vice Chair	Cleveland Clinic Foundation, Cardiovascular Medicine—Medical Director, Noninvasive Vascular Laboratory	None	None	Summit Doppler Systems Zin Medical	AstraZeneca Theravasc	None	None	3.1, 3.2, 5.1–5.3, and 5.6.
Coletta Barrett	Our Lady of the Lake Regional Medical Center—Vice President	None	None	None	None	None	None	None
Neal R. Barshes	Baylor College of Medicine, Division of Vascular Surgery and Endovascular Therapy Michael E. DeBakey Department of Surgery— Assistant Professor	None	None	None	None	None	None	None
Matthew A. Corriere	University of Michigan— Frankel Professor of Cardiovascular Surgery, Associate Professor of Surgery	None	None	None	None	None	None	None
Douglas E. Drachman	Massachusetts General Hospital—Training Director	Abbott Vascular St. Jude Medical	None	None	Atrium Medical Bard Lutonix	None	None	4, 8.1.1– 9.1.2, and 10.2.2.
Lee A. Fleisher	University of Pennsylvania Health System Department of Anesthesiology and Critical Care—Chair	None	None	None	None	None	None	None
Francis Gerry R. Fowkes	University of Edinburgh— Emeritus Professor of Epidemiology	AstraZeneca† Bayer Merck	None	None	None	None	None	5.1–5.3, 5.6, 5.10, 7, and 9.2.
Naomi M. Hamburg	Boston University School of Medicine, Cardiovascular Medicine Section—Associate Professor of Medicine	None	None	None	None	None	None	None
Scott Kinlay	VA Boston Healthcare System—Associate Chief, Cardiology Director, Cardiac Catheterization Laboratory & Vascular Medicine	None	None	None	Medtronic† The Medicines Company†	None	None	4, 5.6, 8.1.1, 9.1.1, 10.2.1 and 10.2.2.
Robert Lookstein	Mount Sinai Medical Center—Chief, Interventional Radiology; Professor of Radiology and Surgery; Vice Chair, Department of Radiology	Boston Scientific Medrad Interventional Possis The Medicines Company	None	Cordis‡	Shockwave (DSMB)	None	None	4, 5.6, 8.1.1, 9.1.1, 10.2.1 and 10.2.2.
Sanjay Misra	Mayo Clinic, Division of Vascular and Interventional Radiology—Professor; Department of Radiology—Interventional Radiologist	None	None	None	Johnson & Johnson (DSMB)	None	None	4, 7, 8, and 10.2.2.
Leila Mureebe	Duke University Medical Center—Associate Professor of Surgery, Division of Vascular Surgery	None	None	None	None	None	None	None
Jeffrey W. Olin	Ichan School of Medicine at Mount Sinai, Zena and Michael A. Wiener Cardiovascular Institute and Marie-Josée and Henry R. Kravis Center for Cardiovascular Health— Professor of Medicine, Cardiology; Director, Vascular Medicine	AstraZeneca Merck Novartis Plurestem	None	Northwind†	AstraZeneca†	None	None	5.1–5.3, 5.6, 5.10, and 12.
Rajan A. G. Patel	John Ochsner Heart & Vascular Center, Ochsner Clinical School, University of Queensland School of Medicine—Senior Lecturer	None	None	None	None	None	None	None
Judith G. Regensteiner	University of Colorado, Health Sciences Center, Division of Cardiology— Associate Professor of Medicine	None	None	None	None	None	None	None
Andres Schanzer	University of Massachusetts Medical School—Professor of Surgery and Quantitative Health Sciences; Program Director, Vascular Surgery Residency	Cook Medical	None	None	None	None	None	4, 8.1.1, 9.1.1 and 10.2.2.
Mehdi H. Shishehbor	Cleveland Clinic, Interventional Cardiology and Vascular Medicine— Director, Endovascular Services	BostonScientific‡ Medtronic‡	None	None	None	AtriumMedical AstraZeneca†	None	4, 8.1.1– 9.1.2, and 10.2.2.
Kerry J. Stewart	Johns Hopkins University, School of Medicine; Johns Hopkins Bayview Medical Center— Professor of Medicine; Director, Clinical and Research Exercise Physiology	None	None	None	None	None	None	None
Diane Treat-Jacobson	University of Minnesota, School of Nursing— Professor	None	None	None	None	None	None	None
M. Eileen Walsh	University of Toledo, College of Nursing— Professor	None	None	None	None	None	None	None

This table represents the relationships of committee members with industry and other entities that were determined to be relevant to this document. These relationships were reviewed and updated in conjunction with all meetings and/or conference calls of the writing committee during the document development process. The table does not necessarily reflect relationships with industry at the time of publication. A person is deemed to have a significant interest in a business if the interest represents ownership of ≥5% of the voting stock or share of the business entity, or ownership of ≥$5000 of the fair market value of the business entity; or if funds received by the person from the business entity exceed 5% of the person's gross income for the previous year. Relationships that exist with no financial benefit are also included for the purpose of transparency. Relationships in this table are modest unless otherwise noted.

* Writing committee members are required to recuse themselves from voting on sections to which their specific relationships with industry and other entities may apply. Section numbers pertain to those in the full-text guideline.

† Significant relationship.

‡ No financial benefit.

AACVPR indicates American Association of Cardiovascular and Pulmonary Rehabilitation; ACC, American College of Cardiology; ACE, Accreditation for Cardiovascular Excellence; AHA, American Heart Association; AMA, American Medical Association; DSMB, data and safety monitoring board; EUCLID, Effects of Ticagrelor and Clopidogrel in Patients with Peripheral Artery Disease; FDA, US Food and Drug Administration; HRS, Heart Rhythm Society; MI, myocardial infarction; NCDR, National Cardiovascular Data Registry; NIH, National Institutes of Health; NHLBI, National Heart, Lung, and Blood Institute; PCORI, Patient-Centered Outcomes Research Institute; PI, primary investigator; PLX-PAD, placental-derived adherent stromal cell; SCAI, Society for Cardiovascular Angiography and Interventions; SCVS, Society for Clinical Vascular Surgery; SIR, Society of Interventional Radiology; SVM, Society for Vascular Medicine; SVN, Society for Vascular Nursing; SVS, Society for Vascular Surgery; TASC, Trans-Atlantic Inter-Society Consensus for the Management of Peripheral Arterial Disease; VA, Veterans Affairs; VESS, Vascular and Endovascular Surgery Society; and VIVA, Vascular Intervention Advances.

## Appendix 2

**Table:** Reviewer Relationships With Industry and Other Entities (Comprehensive)—2016 AHA/ACC Guideline on the Management of Patients With Lower Extremity Peripheral Artery Disease (March 2016)

Reviewer	Representation	Employment	Consultant	Speakers Bureau	Ownership/ Partnership/ Principal	Personal Research	Institutional, Organizational, or Other Financial Benefit	Expert Witness
Deepak L. Bhatt	Official Reviewer—ACC Board of Trustees	Brigham and Women's Hospital— Executive Director of Interventional Cardiovascular Programs; Harvard Medical School—Professor of Medicine	Elsevier	None	None	Amarin* Amgen* AstraZeneca* Bristol-Myers Squibb* Cardax† Eisai* Ethicon* FlowCo† Forest Laboratories* Ischemix* Mayo Clinic Medtronic* Merck† Pfzer* PLx Pharma† Regado Biosciences† Roche* Sanof-aventis* St. Jude Medical Takeda† The Medicines Company* WebMD*	Belvoir Publications (Editor)* Biotronik Boston Scientific Clinical Cardiology (Deputy Editor)† Harvard Clinical Research Institute HMP Communications (Editor)* Duke Clinical Research Institute* Journal of Invasive Cardiology (Editor)* Medscape Cardiology Slack Publications (Editor)* St. Jude Medical VA Healthcare System†	None
Mark A. Creager	Official Reviewer—AHA	Dartmouth-Hitchcock Medical Center—Director	None	None	None	None	AHA (Past President)†	None
Philip Goodney	Official Reviewer—AHA	Dartmouth-Hitchcock— Associate Professor of Surgery and The Dartmouth Institute Director	None	None	None	NIH*	NIH	None
John S. Ikonomidis	Official Reviewer— ACC/AHA Task Force on Clinical Practice Guidelines	Medical University of South Carolina— Chief	None	None	None	None	None	None
Amy W. Pollak	Official Reviewer—AHA	Mayo Clinic— Cardiovascular Medicine Physician	None	None	None	None	None	None
Michael D. White	Official Reviewer—ACC Board of Governors	Catholic Health Initiatives—Chief Academic Officer	Anthera Pharmaceuticals†	None	None	AstraZeneca†	None	None
Ehrin J. Armstrong	Organizational Reviewer— SVM	University of Colorado—Director, Interventional Cardiology	Abbott Medtronic Merck Spectranetics	None	None	None	None	None
Bernadette Aulivola	Organizational Reviewer—VESS	Loyola University medical Center, Stritch School of Medicine—Director, Division of Vascular Surgery and Endovascular Therapy; Associate Professor, Department of Surgery; Program Director, Vascular Surgery Fellowship; Medical Director, Vascular Noninvasive lab	None	None	None	None	None	None
Alison Bailey	Organizational Reviewer—AACVPR	University of Tennessee Chattanooga— Cardiologist	None	None	None	CSL Behring	AACVPR† ZOLL Medical	None
Todd Brown	Organizational Reviewer—AACVPR	University of Alabama at Birmingham— Associate Professor	None	None	None	Amgen* Omthera† NIH*	None	None
Kristen Columbia	Organizational Reviewer—SVN	University of Maryland Baltimore Washington Medical Center, Maryland Vascular Center— Nurse practitioner	None	None	None	None	None	None
Michael S. Conte	Organizational Reviewer—SVS	University of California San Francisco— Professor and Chief	Cook Medical Medtronic	None	None	Bard	University of California Department of Surgery	None
Alik Farber	Organizational Reviewer—SCVS	Boston Medical Center—Chief, Division of Vascular Surgery	Bard†	None	None	None	None	None
Robert Feezor	Organizational Reviewer—VESS	University of Florida—Associate Professor of Surgery, Division of Vascular Surgery and Endovascular Therapy	Cook Medical* Medtronic Terumo	None	None	Cook Medical	Cook Medical Novate	Defendant, peripheral angioplasty, 2015
Dmitriy N. Feldman	Organizational Reviewer—SCAI	Weill Cornell Medical College, New York Presbyterian Hospital—Associate Professor of Medicine	AstraZeneca	Abbott Bristol-Myers Squibb† Daiichi-Sankyo Eli Lilly Medtronic Pfzer The Medicines Company	None	None	Biotronic The Medicines Company	None
Jonathan Golledge	Organizational Reviewer—TASC	James Cook University— Professor, Department of Surgery, Head of Vascular Biology Unit	None	None	None	James Cook University*	None	None
Bruce H. Gray	Organizational Reviewer—SCAI	Greenville Health System—Director of Clinical Trials, Department of Surgery	None	Medtronic†	None	Abbott† W.L. Gore†	NCDR† ACC†	None
William R. Hiatt	Organizational Reviewer—TASC	Colorado Prevention Center—Professor of Medicine	None	None	None	AstraZeneca* Bayer* CSI Kowa Kyushu University Merck Pluristem* ReNeuron	CPC Clinical Research* NIH*	None
Joseph Mills	Organizational Reviewer—SVS	Baylor College of Medicine— Professor and Chief, Division of Vascular surgery and Endovascular Therapy	None	None	None	None	AnGes Bayer Cesca	None
Mohammad Reza Rajebi	Organizational Reviewer—SIR	University of Colorado Denver— Assistant Professor	None	None	None	None	None	None
Mitchell J. Silver	Organizational Reviewer—SVM	McConnell Heart Hospital for Critical Limb Care— Director of Vascular Imaging	Boston Scientific W.L. Gore Medtronic	Bristol-Myers Squibb* Pfzer*	Contego Medical*	None	W.L. Gore Medtronic NIH	None
Lily Thomson	Organizational Reviewer—SVN	Hôpital St-Boniface Hospital—Clinical Research Coordinator, Vascular Surgery Nurse, Section of Vascular Surgery, Health Sciences Centre	None	None	None	None	None	None
Sana M. Al-Khatib	Content Reviewer— ACC/AHA Task Force on Clinical Practice Guidelines	Duke Clinical Research Institute— Associate Professor of Medicine	None	None	None	FDA* NHLBI* PCORI* VA (DSMB)	HRS (Board of Trustees)† Elsevier*	None
Herbert Aronow	Content Reviewer—ACC Peripheral Vascular Disease Member Section	Rhode Island Hospital—Director of Cardiac Catheterization Laboratories	None	None	None	Silk Road Medical† Saint Luke's Health System The Medicines Company†	Bard NIH PCORI† SVM† W.L. Gore	
Joshua A. Beckman	Content Reviewer	Vanderbilt University Medical Center— Director	AstraZeneca* Merck* Sanof*	None	EMX† JanaCare†	Bristol-MyersSquibb* Merck* NIH	Vascular Interventional Advances	Defendant, venous thrombo-embolism, 2015*
James C. Blankenship	Content Reviewer	Geisinger Medical Center—Staff Physician; Director, Cardiac Catheterization Laboratory	None	None	None	Abbott† AstraZeneca† Boston Scientific† GlaxoSmithKline† Hamilton Health Sciences† Medinal LTD† Orexigen Therapeutics† St. Jude Medical† Stentys† Takeda Pharmaceuticals†	SCAI (PastPresident)† AMA†	None
Biykem Bozkurt	Content Reviewer— ACC/AHA Task Force on Clinical Practice Guidelines	Michael E. DeBakey VA Medical Center—The Mary and Gordon Cain Chair and Professor of Medicine	None	None	None	Novartis	None	None
Joaquin E. Cigarroa	Content Reviewer— ACC/AHA Task Force on Clinical Practice Guidelines	Oregon Health and Science University—Clinical Professor of Medicine	None	None	None	None	ACC/AHA† AHA† ASA† Catheterization and Cardiovascular Intervention† Portland Metro Area AHA(President)† SCAI Quality Interventional Council† NIH	None
Federico Gentile	Content Reviewer— ACC/AHA Task Force on Clinical Practice Guidelines	Centro Medico Diagnostico— Director, Cardiovascular Disease	None	None	None	None	None	None
Anuj Gupta	Content Reviewer—ACC Peripheral Vascular Disease Member Section	University of Maryland— Assistant Professor of Medicine	None	None	None	Seimens* Medtronic†	Direct Flow Medical† Edwards†	None
John Jeb Hallett	Content Reviewer	Medical University of South Carolina— Clinical Professor of Surgery	None	None	None	None	None	None
Alan Hirsch	Content Reviewer	University of Minnesota Medical School—Professor of Medicine, Epidemiology and Community Health, and Director Vascular Medicine Program	Merck* Novartis†	None	None	Bayer* Pluristem (PLX-PAD trial–PI)† AstraZeneca (EUCLID trial–PI)† Pluristem*	AHA† Tactile Medical*	None
Mark A. Hlatky	Content Reviewer— ACC/AHA Task Force on Clinical Practice Guidelines	Stanford University School of Medicine— Professor of Health Research and Policy, Professor of Medicine	Acumen* Genentech	None	None	Blue Cross/Blue Shield Center for Effectiveness Evaluation* George Institute HeartFlow* NHLBI Sanofi-aventis	ACC (Associate Editor)*	None
Michael R. Jaff	Content Reviewer	Newton-Wellesley Hospital; Harvard Medical School— Professor of Medicine	AOPA Cardinal Health Covidien† Micell Vascular Therapies	None	MC10† Janacare† Northwind PQ Bypass Primacea SanoV Valiant Medical	Abbott† Boston Scientific† Cordis† IC Sciences Medtronic† Novello	CBSET Intersocietal Accreditation Commission SCAI† VIVA Physicians Group*	None
José A. Joglar	Content Reviewer— ACC/AHA Task Force on Clinical Practice Guidelines	UT Southwestern Medical Center— Professor of Internal Medicine; Clinical Cardiac Electrophysiology— Fellowship Program Director	None	None	None	None	None	None
Glenn N. Levine	Content Reviewer— ACC/AHA Task Force on Clinical Practice Guidelines	Baylor College of Medicine— Professor of Medicine; Director, Cardiac Care Unit	None	None	None	None	None	None
Khusrow Niazi	Content Reviewer— ACC Peripheral Vascular Disease Member Section	Emory University Department of Medicine— Associate Professor of Medicine	None	Medtronic*	None	Bard Impeto Terumo	None	Plaintiff, MI resulting in death, 2015*
Paul D. Varosy	Content Reviewer—Task Force on Performance Measures	VA Eastern Colorado Health Care System—Associate Professor	None	None	None	VA Health Services Research and Development (PI)*	AHA (Guest Editor)†	None
Christopher J. White	Content Reviewer	Ochsner Clinical School, University of Queensland— Chairman, Department of Cardiology	Neovasc	None	None	AstraZeneca Pharmaceuticals NIH Neovasc Surmodics	ACE (Board of Directors)†	None

This table represents all relationships of reviewers with industry and other entities that were reported by authors, including those not deemed to be relevant to this document, at the time this document was under development. The table does not necessarily reflect relationships with industry at the time of publication. A person is deemed to have a significant interest in a business if the interest represents ownership of ≥5% of the voting stock or share of the business entity, or ownership of ≥$5000 of the fair market value of the business entity; or if funds received by the person from the business entity exceed 5% of the person's gross income for the previous year. Relationships that exist with no financial benefit are also included for the purpose of transparency. Relationships in this table are modest unless otherwise noted. Please refer to http://www.acc.org/guidelines/about-guidelines-and-clinical-documents/relationships-with-industry-policy for definitions of disclosure categories or additional information about the ACC/AHA Disclosure Policy for Writing Committees.

* Significant relationship.

† No financial benefit.

AACVPR indicates American Association of Cardiovascular and Pulmonary Rehabilitation; ACC, American College of Cardiology; ACE, Accreditation for Cardiovascular Excellence; AHA, American Heart Association; AMA, American Medical Association; DSMB, data and safety monitoring board; EUCLID, Effects of Ticagrelor and Clopidogrel in Patients with Peripheral Artery Disease; FDA, US Food and Drug Administration; HRS, Heart Rhythm Society; MI, myocardial infarction; NCDR, National Cardiovascular Data Registry; NIH, National Institutes of Health; NHLBI, National Heart, Lung, and Blood Institute; PCORI, Patient-Centered Outcomes Research Institute; PI, primary investigator; PLX-PAD, placental-derived adherent stromal cell; SCAI, Society for Cardiovascular Angiography and Interventions; SCVS, Society for Clinical Vascular Surgery; SIR, Society of Interventional Radiology; SVM, Society for Vascular Medicine; SVN, Society for Vascular Nursing; SVS, Society for Vascular Surgery; TASC, Trans-Atlantic Inter-Society Consensus for the Management of Peripheral Arterial Disease; VA, Veterans Affairs; VESS, Vascular and Endovascular Surgery Society; and VIVA, Vascular Intervention Advances.

## Appendix 3

**Table:** Abbreviations

AAA = abdominal aortic aneurysm
ABI = ankle-brachial index
ALI = acute limb ischemia
CLI = critical limb ischemia
GDMT = guideline-directed management and therapy
MRA = magnetic resonance angiography
PAD = peripheral artery disease
RCT = randomized controlled trial
SPP = skin perfusion pressure
TBI = toe-brachial index
TcPO_2_ = transcutaneous oxygen pressure
QoL = quality of life
